# Adaptive immune responses to two-dose COVID-19 vaccine series in healthy Canadian adults ≥ 50 years: a prospective, observational cohort study

**DOI:** 10.1038/s41598-024-59535-0

**Published:** 2024-04-18

**Authors:** Gabrielle N. Gaultier, Brynn McMillan, Chad Poloni, Mandy Lo, Bing Cai, Jean J. Zheng, Hannah M. Baer, Hennady P. Shulha, Karen Simmons, Ana Citlali Márquez, Sofia R. Bartlett, Laura Cook, Megan K. Levings, Theodore Steiner, Inna Sekirov, James E. A. Zlosnik, Muhammad Morshed, Danuta M. Skowronski, Mel Krajden, Agatha N. Jassem, Manish Sadarangani

**Affiliations:** 1 Department of Pediatrics, University of British Columbia, 950 West 28th Avenue, Vancouver, BC V5Z 4H4 Canada; 2 Vaccine Evaluation Center, British Columbia Children’s Hospital Research Institute, Vancouver, BC Canada; 3 Experimental Medicine Program, University of British Columbia, Vancouver, BC Canada; 4 Department of Microbiology and Immunology, University of British Columbia, Vancouver, BC Canada; 5 British Columbia Children’s Hospital Research Institute, University of British Columbia, Vancouver, BC Canada; 6 Institute of Infection, Inflammation & Immunity, College of Medical, Veterinary and Life Sciences, University of Glasgow, Glasgow, UK; 7 British Columbia Centre for Disease Control, Vancouver, BC Canada; 8 School of Population and Public Health, University of British Columbia, Vancouver, BC Canada; 9 Department of Medicine, University of British Columbia, Vancouver, BC Canada; 10 Department of Microbiology & Immunology, University of Melbourne at the Peter Doherty Institute for Infection and Immunity, Melbourne, VIC Australia; 11 Department of Surgery, University of British Columbia, Vancouver, BC Canada; 12 School of Biomedical Engineering, University of British Columbia, Vancouver, BC Canada; 13 Department of Pathology and Laboratory Medicine, University of British Columbia, Vancouver, BC Canada

**Keywords:** Immunology, Vaccines

## Abstract

To evaluate immune responses to COVID-19 vaccines in adults aged 50 years and older, spike protein (S)-specific antibody concentration, avidity, and function (via angiotensin-converting enzyme 2 (ACE2) inhibition surrogate neutralization and antibody dependent cellular phagocytosis (ADCP)), as well as S-specific T cells were quantified via activation induced marker (AIM) assay in response to two-dose series. Eighty-four adults were vaccinated with either: mRNA/mRNA (mRNA-1273 and/or BNT162b2); ChAdOx1-S/mRNA; or ChAdOx1-S/ChAdOx1-S. Anti-S IgG concentrations, ADCP scores and ACE2 inhibiting antibody concentrations were highest at one-month post-second dose and declined by four-months post-second dose for all groups. mRNA/mRNA and ChAdOx1-S/mRNA schedules had significantly higher antibody responses than ChAdOx1-S/ChAdOx1-S. CD8^+^ T-cell responses one-month post-second dose were associated with increased ACE2 surrogate neutralization. Antibody avidity (total relative avidity index) did not change between one-month and four-months post-second dose and did not significantly differ between groups by four-months post-second dose. In determining COVID-19 correlates of protection, a measure that considers both antibody concentration and avidity should be considered.

## Introduction

The most widely used coronavirus disease 2019 (COVID-19) vaccines in Canada have been the mRNA formulations BNT162b2 and mRNA-1273, and the viral vector vaccine ChAdOx1-S^1–4^. Initial recommendations included an increased interval between first and second doses of up to 16 weeks and permissive use of mixed product regimens to address relatively limited early vaccine supply^5,6^. Older individuals have been prioritized for vaccination due to their increased risk for severe COVID-19 complications. Adults aged 50 years and older may be at increased risk^7^ due to decreased immune function caused by immunological aging (immunosenescence)^8^ and/or chronic low grade systemic inflammation (inflammaging)^9^.

There are currently no accepted surrogates or correlates of protection (CoP) against symptomatic infection or severe disease for COVID-19. Antigen-specific antibody concentrations have been suggested as a practical CoP. However, antibody concentrations alone do not provide information on the functional abilities of antigen-specific antibodies and may not correlate with antibody function or T-cell responses^10^. The objective of this study was to evaluate adaptive immune responses during a two-dose series of COVID-19 vaccines in community-dwelling, immunocompetent adults aged ≥ 50 years. Concentrations of anti-spike protein (S) IgG (S-IgG) were measured as well as antibody function through S-IgG avidity, surrogate neutralization via angiotensin-converting enzyme 2 (ACE2) inhibiting antibody concentrations, and antibody dependent cellular phagocytosis (ADCP). A small sub-group of participants was also selected for quantification of S-specific T cells.

## Results

### Study population

**Figure 1:**
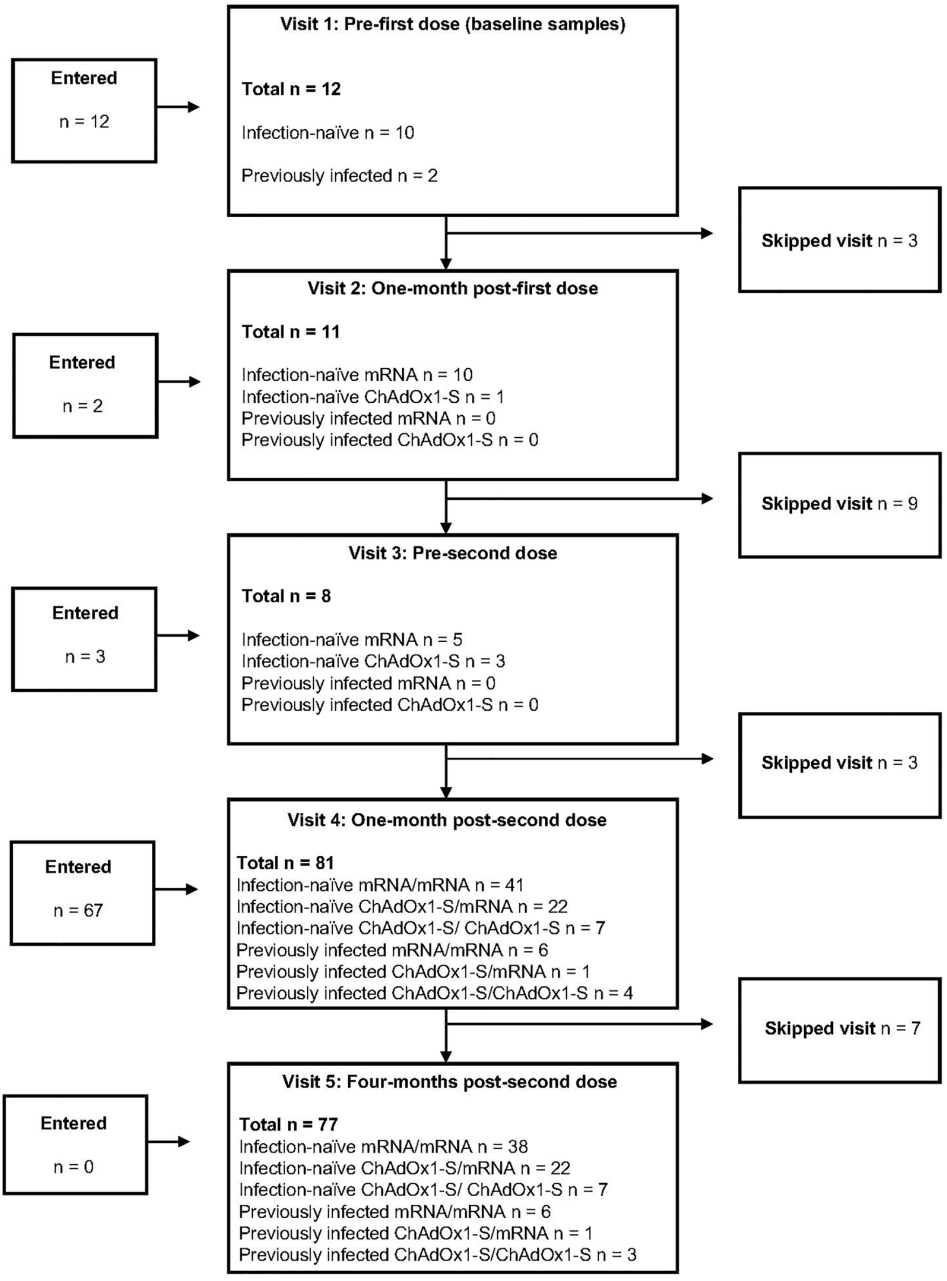
Prospective evaluation of immunity after COVID-19 vaccines (PREVENT-COVID) study CONSORT diagram. Entered study is defined as the first visit a sample was obtained for testing from a participant. Infection-naïve participants have not received a positive COVID-19 PCR test or tested positive on clinical testing assays while previously infected participants received a positive COVID-19 PCR test or tested positive on clinical testing assays. The mRNA participants received either mRNA-1273 or BNT162b2 as a first dose, mRNA/mRNA participants were vaccinated with a combination of mRNA-1273/mRNA-1273, BNT162b2/BNT162b2, or BNT162b2/mRNA-1273. ChAdOx1-S/mRNA participants received either mRNA-1273 or BNT162b2 for their second doses.

The study included 84 participants in five study visits spanning April to December 2021, with a median age of 61 years (Table 1), (64 years for the T cell cohort, Supplementary table 1). Most participants (58.0%) received two mRNA vaccines, including homologous (same vaccines) and heterologous (different vaccines) combinations (Fig. 1). Interval between vaccine doses ranged from 7 to 15 weeks. Most participants identified as White (85.7%) and female (61.9%), being in very good (39.3%) or excellent (32.1%) health. By the last study visit (four-months post-second dose), 13% of participants had acquired a lab-confirmed severe acute respiratory syndrome coronavirus 2 (SARS-CoV-2) infection (Fig. 1).

**Table 1:** Participant demographics.

		Infection-naïve participants (completed two-dose COVID-19 vaccine series)		
	All participants	mRNA/mRNA	ChAdOx1-S/mRNA	ChAdOx1-S/ChAdOx1-S
n	84	42	22	7
Age (years) mean, median, range, interquartile range	62, 61, 50 – 83, 55.8—67	66, 67, 51–83, 61–71	57, 56, 51–64, 53–60	58, 60, 51–63, 56–61
Sex n (%)	Female = 52 (61.9) Male = 32 (38.1)	Female = 27 (64.3) Male = 15 (35.7)	Female = 13 (59.1) Male = 9 (40.9)	Female = 5 (71.4) Male = 2 (28.6)
Gender	Man = 32 (38.1) Woman = 50 (59.5) Non-binary = 2 (2.4)	Man = 15 (35.7) Woman = 27 (64.3) Non-binary = 0 (0)	Man = 9 (40.91) Woman = 12 (54.5) Non-binary = 1 (4.5)	Man = 2 (28.6) Woman = 4 (57.1) Non-binary = 1 (14.3)
BMI Mean, median (range)	26.4, 26.3 (18.2 – 43.6)	27.1, 27.3, 18.2–43.6	26.1, 24.9, 19.5–36.4	24, 23.2, 20.3–29.1
Ethnicity n (%)	White = 72 (85.7)	White = 36 (85.7)	White = 20 (90.9)	White = 6 (85.7)
	Chinese = 6 (7.1)	Chinese = 3 (7.1)	Chinese = 2 (9.1)	Chinese = 0 (0)
	White and Indigenous = 2 (2.4)	White and Indigenous = 0 (0)	White and Indigenous = 0 (0)	White and Indigenous = 1 (14.3)
	South Asian = 2 (2.4)	South Asian = 1 (2.4)	South Asian = 0 (0)	South Asian = 0 (0)
	Korean = 1 (1.2)	Korean = 0 (0)	Korean = 0 (0)	Korean = 0 (0)
	Persian = 1 (1.2)	Persian = 0 (0)	Persian = 0 (0)	Persian = 0 (0)
Health Status n (%)	Excellent = 27 (32.1)	Excellent = 8 (19.1)	Excellent = 11 (50.0)	Excellent = 5 (71.4)
	Very Good = 33 (39.3)	Very Good = 20 (47.6)	Very Good = 7 (31.8)	Very Good = 1 (14.3)
	Good = 18 (21.4)	Good = 9 (21.4)	Good = 4 (18.2)	Good = 1 (14.3)
	Fair = 5 (6.0)	Fair = 4 (9.5)	Fair = 0 (0)	Fair = 1 (14.3)
	Mildly Poor = 1 (1.2)	Mildly Poor = 1 (2.4)	Mildly Poor = 0 (0)	Mildly Poor = 0 (0)
Vaccine interval (weeks) mean, median (range)	10, 10 (7—15)	10.6, 10.5 (7 – 15)	10.5, 10.5 (8 – 13)	9.86, 10 (9 – 12)
First vaccine n	BNT162b2 = 43	BNT162b2 = 36	–	–
	mRNA-1273 = 7	mRNA-1273 = 6	–	–
	ChAdOx1-S = 34	–	ChAdOx1-S = 22	ChAdOx1-S = 7
Second vaccine n	BNT162b2 = 43	BNT162b2 = 32	BNT162b2 = 6	–
	mRNA-1273 = 28	mRNA-1273 = 10	mRNA-1273 = 16	–
	ChAdOx1-S = 11	–	–	ChAdOx1-S = 7
	None = 2	–	–	–
Vaccine series* (Infection-naïve) n	mRNA/mRNA = 42	–	–	–
	ChAdOx1-S/mRNA = 22	–	–	–
	ChAdOx1-S/ChAdOx1-S = 7	–	–	–
Vaccine series* (previously infected) n	mRNA/mRNA = 6	–	–	–
	ChAdOx1-S/mRNA = 1	–	–	–
	ChAdOx1-S/ChAdOx1-S = 4	–	–	–

*Infection status by four-months post-dose two.

### Concentrations of spike protein-specific IgG

Although samples were collected at five timepoints, statistical analyses focused on post-second dose responses. These comparisons were completed for infection-naïve participants, which compared each group between one- and four-months post-second dose (Welch’s t-test) as well as all three groups at both post-second dose timepoints (One-way ANOVA with Tukey–Kramer post-hoc). A Welch’s t-test was used to compare infection-naïve and previously infected participants that received the same vaccine series post-second dose. A Bonferroni correction was applied to all t-tests.

**Figure 2:**
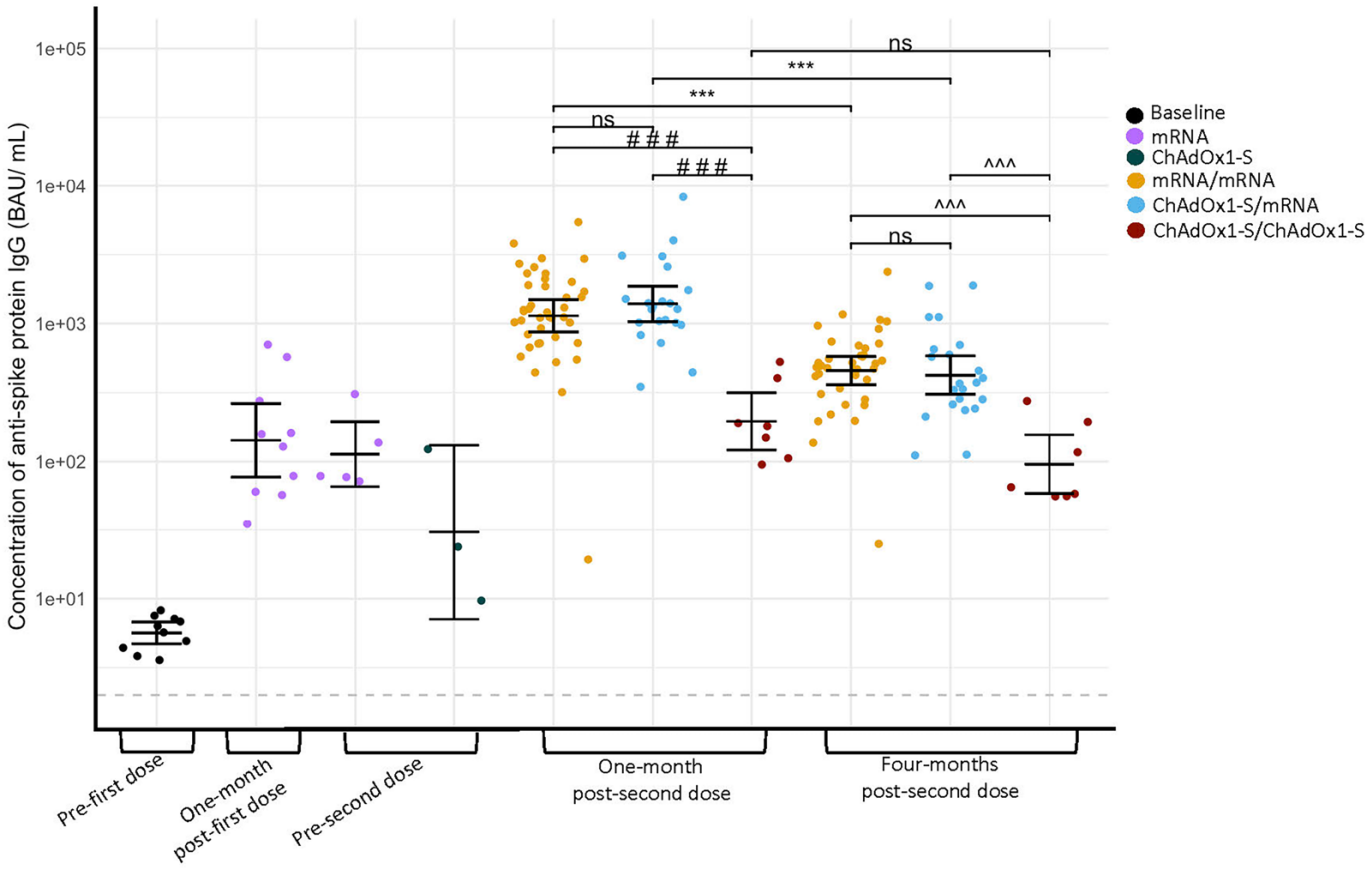
Geometric mean concentrations (GMC) of anti-spike protein IgG in binding antibody units per milliliter (BAU/mL) of infection-naïve participants grouped based on vaccine series. The GMC is represented by the solid line. Data were log_10_ transformed prior to statistical analyses. The grey dashed line represents the lower limit of quantification (LLQ) of 2 BAU/mL, values below the LLQ were assigned a value of 1 BAU/mL for statistical purposes. *** *P* < 0.001, compared concentrations of anti-spike protein IgG between study visits within the same group (Welch’s t-test, a Bonferroni correction was applied adjusting the *P*-values by multiplying by the number of comparisons (seven)). ###*P* < 0.001, compared concentrations of anti-spike protein IgG between groups at one-month post-second dose (One-way ANOVA, Tukey–Kramer post-hoc). ^^^*P* < 0.001, compared concentrations of anti-spike protein IgG between groups at four-months post-second dose (One-way ANOVA, Tukey–Kramer post-hoc). Not significant (ns) *P* > 0.05. Baseline (n = 10), one-month post-first dose (mRNA n = 10), pre-second dose (mRNA n = 5) (ChAdOx1-S n = 3), one-month post-second dose (mRNA/mRNA n = 41) (ChAdOx1-S/mRNA n = 22) (ChAdOx1-S/ChAdOx1-S n = 7), four-months post-second dose (mRNA/mRNA n = 38) (ChAdOx1-S/mRNA n = 22) (ChAdOx1-S/ChAdOx1-S n = 7).

Among infection-naïve participants, geometric mean concentrations (GMCs) of S-IgG (binding antibody units per milliliter (BAU/mL)) (Fig. 2 and Supplementary Fig. 1) were highest at one-month post-second dose, and decreased by four-months post-second dose for mRNA/mRNA (1137 vs. 455 BAU/mL, *P* < 0.001) and ChAdOx1-S/mRNA (1388 vs. 423 BAU/mL, *P* < 0.001), but not ChAdOx1-S/ChAdOx1-S (195 vs. 96 BAU/mL, *P* = 0.442). Significance was not reached for ChAdOx1-S/ChAdOx1-S though similar trends were seen as mRNA/mRNA and ChAdOx1-S/mRNA likely due to smaller group size. Due to the rapid rollout of COVID-19 vaccines in this age group, small numbers of participants were recruited prior to receiving a second dose. For reference, baseline GMC was 6 BAU/mL. One-month post-first dose of either mRNA vaccine was 142 BAU/mL, no samples were collected from ChAdOx1-S participants at this timepoint. Pre-second dose, GMCs consisted of 113 BAU/mL for participants that received a mRNA vaccine and 30 BAU/mL for participants that received ChAdOx1-S. Vaccine series were also compared post-second dose. At one-month and four-months post-second dose, GMCs were higher for mRNA/mRNA and ChAdOx1-S/mRNA groups than ChAdOx1-S/ChAdOx1-S (all comparisons *P* < 0.001). mRNA/mRNA and ChAdOx1-S/mRNA resulted in similar GMCs at both one-month (*P* = 0.627) and four-months post-second dose (*P* = 0.926).

Next, previously infected and infection-naïve participants were compared post-second dose. mRNA/mRNA participants previously infected with SARS-CoV-2 had higher GMCs than infection-naïve participants at one-month (3391 vs. 1137 BAU/mL, *P* = 0.007) and four-months (1615 vs. 455 BAU/mL, *P* < 0.030) post-second dose. With smaller sample size, statistical significance was not reached when previously infected participants and infection-naïve ChAdOx1-S/ChAdOx1-S recipients GMCs were compared one-month (560 vs. 195 BAU/mL, *P* = 1.0) or four-months (220 vs. 96 BAU/mL, *P* = 1.0) post-second dose but appeared to follow a similar trend as mRNA/mRNA. No comparisons could be made between previously infected and infection-naïve ChAdOx1-S/mRNA participants because the minimum number of participants previously infected was too low to perform statistical analyses.

### Total relative avidity index

**Figure 3:**
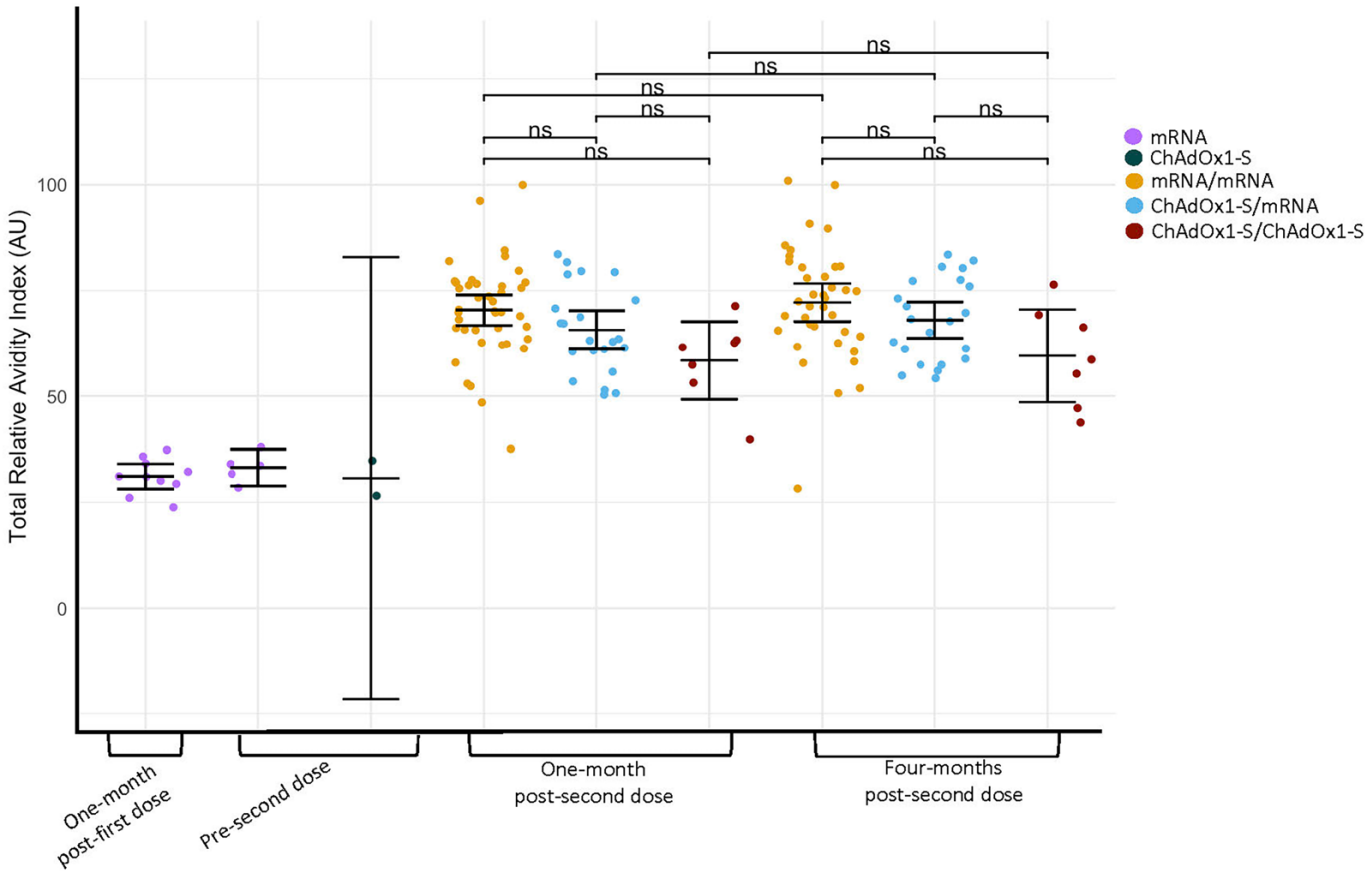
Anti-spike protein specific IgG avidity of infection-naïve participants grouped based on vaccine series. Total Relative Avidity Index (TRAI) of anti-spike protein IgG in arbitrary units (AU); mean TRAI is represented by the solid line. Data were log_10_ transformed prior to statistical analyses. Concentrations of anti-spike protein IgG between study visits within the same group were compared (Welch’s t-test, a Bonferroni correction was applied adjusting the P-values by multiplying by the number of comparisons (seven)). Concentrations of anti-spike protein IgG between groups at one-month and four-months post-second dose (One-way ANOVA, Tukey–Kramer post-hoc). Not significant (ns) *P* > 0.05. One-month post-first dose (mRNA n = 10), pre-second dose (mRNA n = 5) (ChAdOx1-S n = 2), one-month post-second dose (mRNA/mRNA n = 41) (ChAdOx1-S/mRNA n = 22) (ChAdOx1-S/ChAdOx1-S n = 7), four-months post-second dose (mRNA/mRNA n = 38) (ChAdOx1-S/mRNA n = 22) (ChAdOx1-S/ChAdOx1-S n = 7).

S-IgG avidity was quantified through the addition of ammonium thiocyanate. Results were reported as total relative avidity index (TRAI) and total absolute avidity levels (TAA). Mean TRAI is a weighted sum of the proportions of antibodies present at each fraction of ammonium thiocyanate (avidity unit, AU) (Fig. 3 and Supplementary Fig. 2). The same statistical approach as described comparing S-IgG concentrations was used to compare responses post-second dose.

Among infection-naïve participants, means were similar at one-month and four-months post-second dose; mRNA/mRNA (70 vs. 72 AU, *P* = 1.0), ChAdOx1-S/mRNA (66 vs. 68 AU, *P* = 1.0), ChAdOx1-S/ChAdOx1-S (58 vs. 60 AU, *P* = 1.0). Baseline TRAI was not able to be measured because anti-S IgG concentrations did not meet the minimum amount required to perform the assay. TRAI at one-month post-first dose for mRNA recipients was 31 AU and 33 AU pre-second dose. Samples were not able to be collected from ChAdOx1-S recipients at one-month post-second dose; TRAI pre-second dose was 31 AU. To determine differences in avidity between two-dose series, TRAIs were compared between vaccine groups. TRAIs of mRNA/mRNA compared with ChAdOx1-S/ChAdOx1-S were higher at one-month (*P* = 0.025) and four-months (*P* = 0.056) post-second dose. Amongst participants that received ChAdOx1-S for a first dose, no differences between ChAdOx1-S/mRNA and ChAdOx1-S/ChAdOx1-S were found at one-month (*P* = 0.250) or four-months (*P* = 0.230) post-second dose. Differences in means were not found between mRNA/mRNA and ChAdOx1-S/mRNA at one-month (*P* = 0.250) or four-months (*P* = 0.629) post-second dose.

In comparison with infection-naïve participants, previously infected mRNA/mRNA participants had a higher mean TRAI at four-months (86 vs.72 AU, *P* = 0.003) but not one-month (76 vs.70 AU, *P* = 0.140) post-second dose. Due to low numbers of ChAdOx1-S/ChAdOx1-S participants, statistical significance was not achieved when comparing responses based on infection history at one-month (65 vs. 58 AU, *P* = 1.0) or four-months (69 vs. 60 AU, *P* = 1.0) post-second dose although trends were similar to when mRNA/mRNA infection-naïve and previously infected participants were compared.

### Total absolute avidity levels

Total absolute avidity levels (TAA) is a weighted sum of the concentrations of S-IgG present at each fraction of ammonium thiocyanate (GMCs) expressed as absolute avidity unit per milliliter (AAU/mL) (Supplementary Fig. 3 and Supplementary table 2). The same statistical approach as described comparing S-IgG concentrations was used to compare responses post-second dose. TAA of infection-naïve participants followed similar trends to S-IgG GMCs. TAAs peaked at one-month post-second dose and decreased by four-months post-second dose for mRNA/mRNA (825 vs. 345 AAU/mL, *P* < 0.001), and ChAdOx1-S/mRNA (905 vs. 304 AAU/mL, *P* < 0.001). No differences between one-month and four-months post-second dose were found for ChAdOx1-S/ChAdOx1-S (86 vs. 60 AAU/mL, *P* = 1.0) but similar trends were observed in mRNA/mRNA and ChAdOx1-S/mRNA. Similarly, to TRAI, baseline TAA was not able to be measured. TAA of mRNA participants was 43 AAU/mL at one-month post-first dose and 28 AAU/mL pre-second dose. ChAdOx1-S TAA pre-second dose was 13 AAU/mL (samples were not able to be collected from this group one-month post-first dose). At both post-second dose timepoints, mRNA/mRNA and ChAdOx1-S/mRNA participants had higher GMCs than ChAdOx1-S/ChAdOx1-S participants (all comparisons *P* < 0.001). No differences were found between mRNA/mRNA and ChAdOx1-S/mRNA at one-month (*P* = 0.925) or four-months (*P* = 0.805) post-second dose.

Previously infected mRNA/mRNA participants had higher GMCs compared with infection-naïve participants at both one-month (2847 vs. 825 AAU/mL, *P* = 0.010) and four-months (1580 vs. 345 AAU/mL, *P* = 0.008) post-second dose. Again, statistical significance was not reached when GMCs were compared between previously infected and infection-naïve ChAdOx1-S/ChAdOx1-S recipients at one-month (354 vs. 86, *P* = 0.702) or four-months (161 vs. 60 AAU/mL,* P* = 1.0) post-second dose although trends were similar to when mRNA/mRNA infection-naïve and previously infected participants were compared.

### Antibody dependent cellular phagocytosis

**Figure 4:**
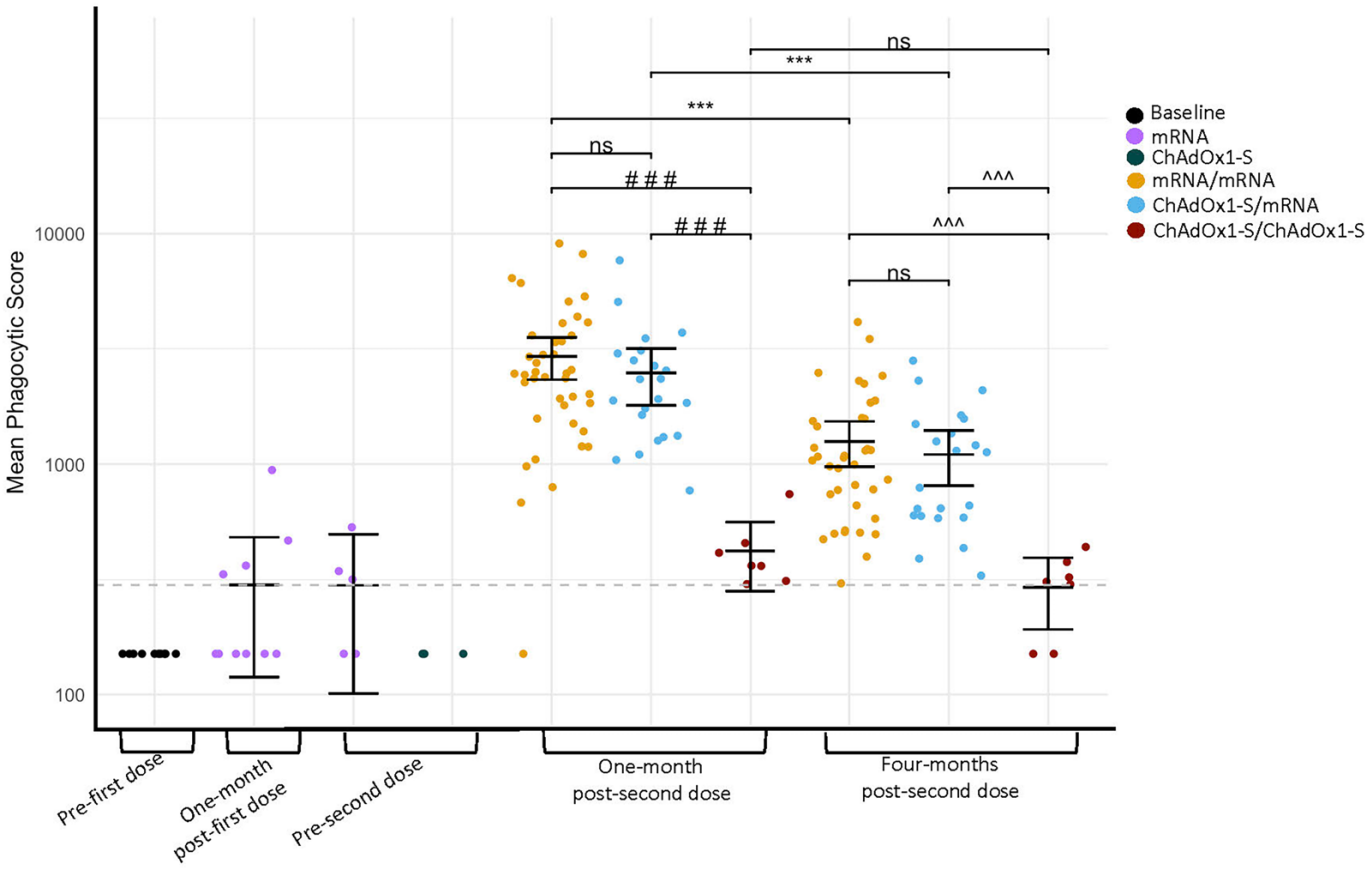
Mean phagocytic scores of anti-spike protein antibodies of infection-naïve participants at each visit based on vaccine series. The mean of the mean phagocytic scores is represented by the solid line with 95% confidence intervals. Data were log_10_ transformed prior to statistical analyses. The grey dashed line represents the lower limit of quantification (LLQ), a mean phagocytic score of 300, values below the LLQ were assigned a value of 150 for statistical purposes. ***P < 0.001, compared concentrations of anti-spike protein antibodies between study visits within the same group (Welch’s t-test, a Bonferroni correction was applied adjusting the *P*-values by multiplying by the number of comparisons (seven)). ###*P* < 0.001, compared concentrations of anti-spike protein antibodies between groups at one-month post-second dose (One-way ANOVA, Tukey–Kramer post-hoc). ^^^*P* < 0.001, compared concentrations of anti-spike protein antibodies between groups at four-months post-second dose (One-way ANOVA, Tukey–Kramer post-hoc). Not significant (ns) *P* > 0.05. Baseline (n = 10), one-month post-first dose (mRNA n = 10), pre-second dose (mRNA n = 5) (ChAdOx1-S n = 3), one-month post-second dose (mRNA/mRNA n = 41) (ChAdOx1-S/mRNA n = 22) (ChAdOx1-S/ChAdOx1-S n = 7), four-months post-second dose (mRNA/mRNA n = 38) (ChAdOx1-S/mRNA n = 22) (ChAdOx1-S/ChAdOx1-S n = 7).

Fc effector function was evaluated via antibody dependent cellular phagocytosis (ADCP), mean phagocytic scores (Fig. 4 and Supplementary table 3). The same statistical approach as described comparing S-IgG concentrations was used to compare responses post-second dose. The mean scores of infection-naïve participants were highest at one-month post-second dose and decreased between one-month and four-months post-second dose for mRNA/mRNA (2936 vs. 1254, *P* < 0.001) and ChAdOx1-S/mRNA (2483 vs. 1102, *P* < 0.001). ChAdOx1-S/ChAdOx1-S participants did not have a difference in scores between one-month and four-months post-second dose (422 vs. 293, *P* = 0.525). The baseline score mean was 150. One-month post-first dose of mRNA, mean score was 301 and 299 pre-second dose. Score pre-second dose of ChAdOx1-S was 150; samples were unable to be collected from these participants at one-month post-first dose. At one-month and four-months post-second dose, mRNA/mRNA and ChAdOx1-S/mRNA had higher scores compared with ChAdOx1-S/ChAdOx1-S (all comparisons *P* < 0.001). No significant difference was found between mRNA/mRNA and ChAdOx1-S/mRNA at one-month (*P* = 0.845) or four-months (*P* = 0.767) post-second dose.

Previously infected mRNA/mRNA participants had higher scores than infection-naïve participants at one-month (6897 vs. 2933, *P* = 0.001) and four-months (4373 vs. 1254, *P* = 0.007) post-second dose. As previously acknowledged, statistical significance was not reached when scores were compared between previously infected and infection-naïve ChAdOx1-S/ChAdOx1-S recipients at one-month (1457 vs. 422, *P* = 0.880) or four-months (1069 vs. 293, *P* = 1.0) post-second dose although trends were similar to when mRNA/mRNA infection-naïve and previously infected participants were compared.

### Angiotensin-converting enzyme 2 inhibiting antibody concentrations

**Figure 5:**
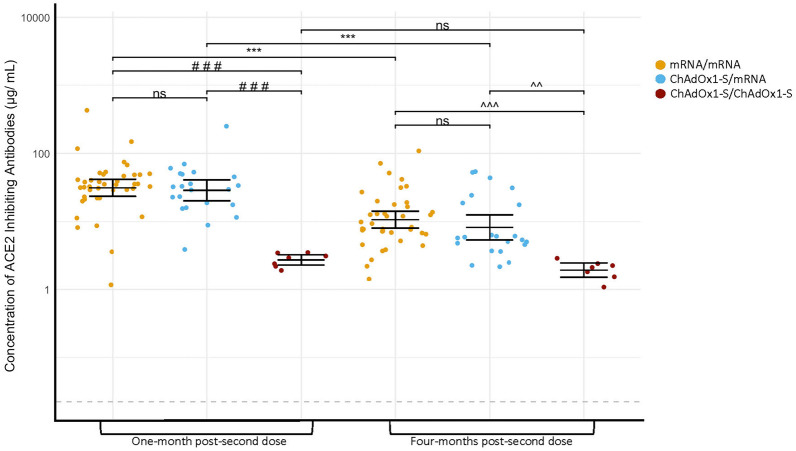
Geometric mean concentrations of ACE2 inhibiting antibodies specific to the SARS-CoV-2 index virus spike protein of infection-naïve participants at one- and four-months post-second dose based on vaccine series. The GMC is represented by the solid line with 95% confidence intervals. Data were log_10_ transformed prior to statistical analyses. The grey dashed line represents the lower limit of quantification (LLQ); values below LLQ were assigned half the value for statistical purposes. Index virus LLQ = 0.02262 μg/mL. ***P < 0.001 compared concentrations of protein specific IgG between the same group at visits 4 and 5 (Welch’s t-test, a Bonferroni correction was applied adjusting the *P*-values by multiplying by the number of comparisons (seven). ###*P* < 0.001, compared concentrations of protein specific IgG between mRNA/mRNA or ChAdOx1-S/mRNA with ChAdOx1-S/ChAdOx1-S at one-month post-second dose (One-way ANOVA, Tukey–Kramer post-hoc). ^^*P* < 0.01, ^^^*P* < 0.001, compared concentrations of protein specific IgG between mRNA/mRNA or ChAdOx1-S/mRNA with ChAdOx1-S/ChAdOx1-S at four-months post-second dose (One-way ANOVA, Tukey–Kramer post-hoc). Not significant (ns) *P* > 0.05. One-month post-second dose (mRNA/mRNA n = 41) (ChAdOx1-S/mRNA n = 22) (ChAdOx1-S/ChAdOx1-S n = 7), four-months post-second dose (mRNA/mRNA n = 39) (ChAdOx1-S/mRNA n = 22) (ChAdOx1-S/ChAdOx1-S n = 7).

Surrogate neutralization was measured via binding of ACE2 to S-coated wells, results reported as concentrations of ACE2 inhibiting antibodies (GMCs, index virus µg/mL; viral variants units/mL) (Fig. 5 (index virus), Supplementary Fig. 4 (a) Alpha/B.1.1.7, (b) Beta/B.1.351, (c) Gamma/P.1, (d) Delta/B.1.617.2, (e) Zeta/P.2, (f) Iota/B.1.526.1, (g) Kappa/B.1.617.1, (h) B.1.617, (i) B.1.617.3 and Supplementary table 4). The same statistical approach as described comparing S-IgG concentrations was used to compare responses post-second dose. GMCs of infection-naïve participants were highest at one-month post-second dose and decreased by four-months post-second dose for the index virus and all variants (all *P* < 0.001) for mRNA/mRNA participants; index (31.3 vs. 10.7 µg/mL) and ChAdOx1-S/mRNA (all *P* < 0.01); index (28.8 vs. 8.3 µg/mL, *P* < 0.001). Concentrations of ACE2 inhibiting antibodies for ChAdOx1-S/ChAdOx1-S participants did not differ between one-month and four-months post-second dose for index virus (2.7 vs. 2.0 µg/mL, *P* = 0.315) or any variants (*P* > 0.05). At one-month and four-months post-second dose, mRNA/mRNA and ChAdOx1-S/mRNA participants had higher concentrations of index virus and nine variant GMCs compared with ChAdOx1-S/ChAdOx1-S; (all comparisons *P* < 0.001 except for ChAdOx1-S/mRNA vs. ChAdOx1-S/ChAdOx1-S at four-months post-second dose for index virus GMCs, *P* = 0.002). There were no significant differences in GMCs (index virus or any variants) between mRNA/mRNA and ChAdOx1-S/mRNA (*P* > 0.05) at one or four-months post-second dose.

GMCs were higher amongst previously infected mRNA/mRNA participants than infection-naïve one-month post-second dose (*P* < 0.05) for Alpha/B.1.1.7, Beta/B.1.351, Gamma/P., and B.1.617.3 but did not differ (*P* > 0.05) for index, Delta/B.1.617.2, Zeta/P.2, Iota/B.1.526.1, Kappa/B.1.617.1 and B.1.617. At four-months post-second dose, mRNA/mRNA previously infected participants had higher ACE2 inhibiting antibody concentrations (*P* < 0.01) against the index virus and all variants than mRNA/mRNA infection-naïve participants. No differences were found between previously infected ChAdOx1-S/ChAdOx1-S and infection-naïve participants at one-month or four-months post-second dose (*P* > 0.05) for index virus or variant GMCs.

### Spike-specific CD4^+^ and CD8^+^ T-cell responses

**Figure 6:**
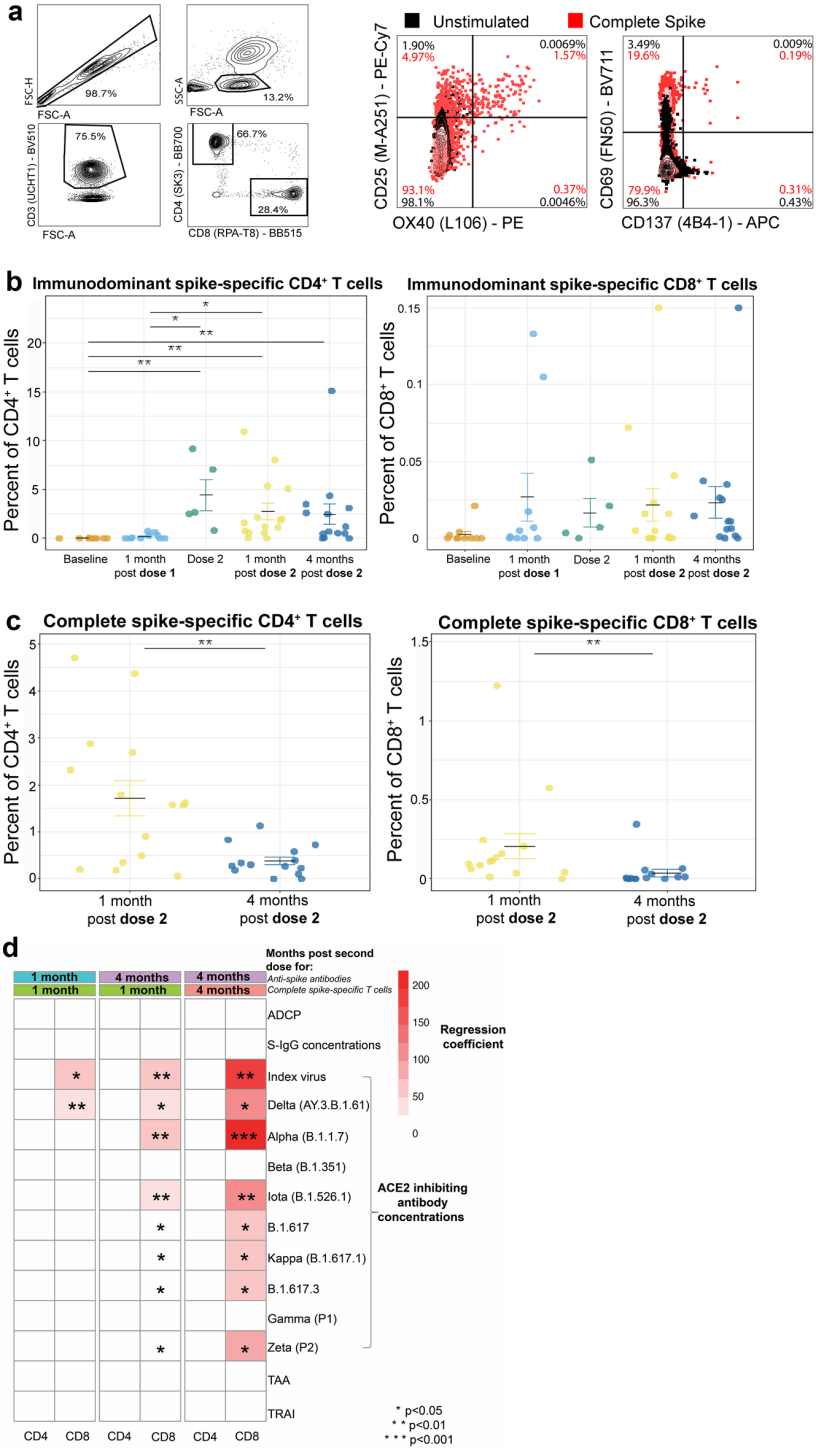
CD4^+^ and CD8^+^ spike-specific T-cell responses significantly decrease by 4 months post dose 2. AIM assays were performed on fresh whole blood following venipuncture. Whole blood was stimulated with immunodominant spike or complete index spike peptide pools for 48 h at 37 °C. The percentages of spike-specific T cells were determined by flow cytometric detection of upregulated co-expression of AIM markers, being CD25 and OX40 for CD4^+^ T cells and CD69 and CD137 for CD8^+^ T cells. Unstimulated wells were used to set AIM + gates and responses in these wells were subtracted as background. (**a**) Representative data from a complete spike antigen stimulated assay at four-months post-second dose. Frequencies of (**b**) immunodominant spike-specific T cells at baseline (n = 11), one-month post-first dose (n = 10), at dose 2 (n = 5), and at one-month (n = 15) and four-months (n = 14) post-second dose and (**c**) complete spike-specific T cells (percent of total CD4^+^ and CD8^+^ T cells) at one-month (n = 15) and four-months (n = 14) post-second dose. (**d**) Multilinear regressions between complete spike specific T cell percentages (from (**b**)) and measures of anti-spike protein IgG (S-IgG) concentration and function (antibody dependent cellular phagocytosis (ADCP), ACE2 inhibiting antibody concentrations, total absolute avidity levels (TAA) and total relative avidity index (TRAI)) at one-month (n = 12) and four-months (n = 12) post-second dose. Regression coefficients are represented by heatmap color and significance by stars. *P < 0.05, **P < 0.01, ***P < 0.001.

A subgroup of participants was selected for activation induced marker (AIM) assays to detect S-specific T cells based on sample availability at study timepoints (n = 18). Whole blood was stimulated with S-antigen for detection of S-specific CD4^+^ T cells (CD25^+^OX40^+^) and CD8^+^ T cells (CD69^+^ and 4-1BB^+^) (Fig. 6a). An overlapping peptide pool covering the immunodominant regions of the S from the index virus was used for earlier timepoints termed immunodominant S (Fig. 6b). Partway through the study, a peptide pool spanning the complete index virus S (complete S) became available which stimulated greater T-cell responses and was used to analyze samples at both post-second dose visits (Fig. 6c).

At one-month post-second dose, all participants had complete S-specific CD4^+^ T cells, while 73.3% had detectable levels of complete spike-specific CD8^+^ T cells. Although reduced in frequency, all participants had detectable complete S-specific CD4^+^ at four-months post-second dose. Frequencies of complete S-specific CD8^+^ reduced by four-months post-second dose (*P* < 0.01), only 42.9% of participants having detectable CD8^+^ T-cell responses.

Linear regression models used frequencies of complete S-specific T cells, S-IgG concentrations, TAA, TRAI, ADCP scores and ACE2 inhibiting antibody concentrations. Complete S-specific CD8^+^ T cells had positive associations with index virus and B.1.617 ACE2 inhibiting antibody concentrations at one-month post-second dose (*P* < 0.01 and *P* < 0.001; Fig. 6d). At four-months post-second dose, complete S-specific CD8^+^ T cells had significant positive associations with index virus ACE2 inhibiting antibody concentrations (*P* < 0.001), as well as Delta/B.1.617.2 (*P* < 0.01), Alpha/B.1.1.7 (*P* < 0.001), Iota/B.1.526.1 (*P* < 0.001), B.1.617 (*P* < 0.05), Kappa/B.1.617.1 (*P* < 0.05), B.1.617.3 (*P* < 0.05), and Zeta/P.2 (*P* < 0.01). To determine if T-cell responses could predict antibody responses, regression models were generated using T cell data from one-month post-second dose and antibody responses from four-months post-second dose. Complete S-specific CD8^+^ T cell frequency positively associated with index virus ACE2 inhibiting antibody concentrations (*P* < 0.001), as well as ACE2 inhibiting antibody concentrations for Delta/B.1.617.2 (*P* < 0.01), Alpha/B.1.1.7 (*P* < 0.001), Iota/B.1.526.1 (*P* < 0.001), B.1.617 (*P* < 0.01), Kappa/B.1.617.1 (*P* < 0.05), B.1.617.3 (*P* < 0.05), and Zeta/P.2 (*P* < 0.01).

### Effects of demographic factors on spike protein-specific antibody responses

To determine which demographic factors influenced antibody responses to vaccination, multivariable linear regression analyses (Table 2) were performed (univariable results, Supplementary table 5). Vaccination with two doses of mRNA/mRNA or ChAdOx1-S/mRNA resulted in higher S-IgG concentrations compared with participants who received ChAdOx1-S/ChAdOx1-S at one-month post-second dose (*P* < 0.001). Similar relationships were identified for TRAI (*P* = 0.014), ADCP (*P* < 0.001) and index virus ACE2 inhibiting antibody concentrations (*P* < 0.001). SARS-CoV-2 previously infected participants had higher S-IgG concentrations (*P* = 0.001) and ADCP scores (*P* < 0.001) compared with infection-naïve participants. Compared with an excellent health status, participants with a health status of very good, good, fair, or mildly poor was associated with higher S-IgG concentrations (*P* = 0.004) and ADCP scores (*P* = 0.001), as well as ACE2 inhibiting antibody concentrations (*P* = 0.011) one-month post-second dose. Body mass index (BMI) of overweight or obese resulted in higher ACE2 inhibiting antibody concentrations (*P* = 0.025) compared to participants with normal or underweight BMI. Shorter vaccine intervals (< 13 weeks) were associated with higher S-IgG (*P* = 0.001) and ACE2 inhibiting antibody concentrations (*P* = 0.002) compared to participants with a longer vaccine interval (≥ 13 weeks). Age, ethnicity, and sex were not found to have a significant impact on humoral immune responses to vaccination. No evidence was found that age, BMI and vaccine interval significantly impacted the magnitude of CD4^+^ and CD8^+^ T-cell responses (Supplementary table 6).

**Table 2:** Multivariable linear regression between either concentration anti-spike protein specific IgG, total relative avidity index (TRAI), antibody dependent cellular phagocytosis (ADCP) scores, or ACE2 inhibiting antibody concentrations specific to the index virus and participant demographic factors one-month post-second dose.

Output	Covariate	Coefficient (95% CI), P-value	*P*-value
Concentration anti-spike protein specific IgG	Vaccine series ChAdOx1-S/ ChAdOx1-S vs. mRNA/mRNA and ChAdOx1-S/mRNA combined	− 0.747 (− 0.965 to − 0.528)	< 0.001
	Vaccine interval (≥ 13 weeks vs. < 13 weeks)	− 0.384 (− 0.597 to − 0.170)	0.001
	Health status (Excellent vs. Very good/Good/Fair/Mildly poor combined)	− 0.226 (− 0.378 to − 0.073)	0.004
	Infection status (Previously infected vs. Infection-naïve)	0.369 (0.157 to 0.580)	0.001
TRAI	Vaccine series ChAdOx1-S/ChAdOx1-S vs. mRNA/mRNA and ChAdOx1-S/mRNA combined	− 0.060 (− 0.108 to − 0.012)	0.014
ADCP scores	Vaccine series ChAdOx1-S/ChAdOx1-S vs. mRNA/mRNA and ChAdOx1-S/mRNA combined	− 0.685 (− 0.860 to − 0.509)	< 0.001
	Health status (Excellent vs. Very good/Good/Fair/Mildly poor combined)	− 0.207 (− 0.330 to − 0.084)	0.001
	Infection status (Previously infected vs. Infection-naïve)	0.419 (0.249 to 0.588)	< 0.001
ACE2 inhibiting antibody concentrations	BMI (Normal and Underweight combined vs. Overweight and Obese combined)	− 0.196 (− 0.367 to − 0.025)	0.025
	Vaccine series ChAdOx1-S/ChAdOx1-S vs. mRNA/mRNA and ChAdOx1-S/mRNA combined	− 0.732 (− 0.974 to − 0.490)	< 0.001
	Vaccine interval (≥ 13 weeks vs. < 13 weeks)	− 0.401 (− 0.646 to − 0.156)	0.002
	Health status (Excellent vs. Very good/Good/Fair/Mildly poor combined)	− 0.240 (− 0.422 to − 0.057),	0.011

Data were log_10_ transformed prior to statistical analyses. Confidence interval (CI). Coefficients from multivariable analyses correspond to log_10_ transformed data.

### Contributions of avidity and concentrations to spike protein-specific antibody function

To further investigate the contributions of both S-IgG concentration and avidity on antibody function, multivariable linear regression analyses were performed between concentrations of S-IgG at each avidity level (referred to as the fractional absolute avidity levels (FAA), reported as GMC, BAU/mL) and antibody function one-month post-second dose. FAA is separated into seven different avidity binding levels: very low, low, low-medium, medium, medium–high, high and very high. This analysis was performed with the intention to identify whether a specific level of avidity antibody was associated with antibody function. Both ADCP scores (Supplementary table 7a) and concentrations of index virus ACE2 inhibiting antibodies (Supplementary table 7b) had positive relationships (all *P* < 0.001) with antibody concentrations for all avidity levels.

### Correlation between spike protein-specific antibody concentration and function

Spearman correlations were performed to determine the relationship between anti-S IgG concentrations and ADCP scores, ACE2 index virus inhibiting antibody concentrations and TRAI at both one-month and four-months post-second dose (Supplementary table 8a). A Bonferroni correction was applied to these analyses. At both one-month and four-months post-second dose, mRNA/mRNA and ChAdOx1-S/mRNA had positive relationships between anti-S IgG concentrations and both ADCP scores (*P* < 0.001) and as well as ACE2 inhibiting antibody concentrations (*P* < 0.001). There was no significant relationship between anti-S IgG concentrations and either ADCP scores or ACE2 inhibiting antibody concentrations at either post-second dose timepoints for ChAdOx1-S/ChAdOx1-S (*P* > 0.05). For all three groups, no significant relationship was found between S-IgG and TRAI for post-second dose (*P* > 0.05).

To evaluate the relationship between antibody concentration, avidity and function, correlation analyses between TAA, ADCP scores and ACE2 index virus inhibiting antibody concentrations were performed for post-second dose visits (Supplementary table 8b.). For both post-second dose timepoints, there was a positive relationship between TAA and both ADCP scores and ACE2 inhibiting antibody concentrations for mRNA/mRNA and ChAdOx1-S/mRNA (*P* < 0.001). No significant relationships were found for ChAdOx1-S/ChAdOx1-S at either timepoint between TAA and ADCP scores or ACE2 inhibiting antibody concentrations (*P* > 0.05).

### Modelling protection against symptomatic infection post-two doses based on literature anti-spike protein IgG concentrations

Nearly all mRNA/mRNA and ChAdOx1-S/mRNA participants met the literature suggested S-IgG CoP against symptomatic infection post-second dose (Supplementary table 9). Using a S-IgG of 154 BAU/mL (vaccine series with an mRNA vaccine) as the putative CoP ^11^, 95.5% of all mRNA/mRNA and 91.3% of all ChAdOx1-S/mRNA participants were considered protected at four-months post-second dose. When applying a CoP of 60 BAU/mL^11^, for all ChAdOx1-S/ChAdOx1-S participants, only 63.6% of participants achieved this CoP at four-months post-second dose. If using a pooled model cut-off (100 BAU/mL)^12^, all participants were evaluated for protection against SARS-CoV-2 infection at four-months post-second dose; 97.7% of mRNA/mRNA, 100% of ChAdOx1-S/mRNA and 50% of ChAdOx1-S/ChAdOx1-S participants were considered protected. If only focusing on infection-naïve participants, proportions were altered to 97.4%, 100%, and 42.9% respectively.

## Discussion

A two-dose series containing at least one mRNA vaccine induced more robust antibody responses than two doses of ChAdOx1-S in this cohort based on concentration and function of S-specific antibodies. Although S-IgG concentration, TAA, ADCP scores and ACE2 inhibition decreased rapidly, TRAI did not significantly differ between post-second dose timepoints. Based on the finding that all FAA avidity levels had a positive relationship with ADCP scores and ACE2 index virus inhibiting antibody concentrations, antibody functions were dependent on concentration and avidity of S-specific antibodies. Over 90% of infection-naïve mRNA/mRNA and ChAdOx1-S/mRNA participants at four-months post-second dose would be considered protected against symptomatic SARS-CoV-2 infection using a CoP of 154 BAU/mL.

Declining S-specific antibody responses following a two-dose COVID-19 vaccine series have been previously reported. The ENFORCE study reported that infection-naïve adults (median age 64 years) who received either two doses of mRNA-1273, BNT162b2 (homologous series) or ChAdOx1-S followed by a mRNA vaccine also experienced a decrease in S-IgG and ACE2 inhibiting antibody concentrations 180 days post-second dose^13^. To our knowledge, no other COVID-19 vaccination studies have expressed antibody avidity as TAA. However, because TAA considers both S-IgG concentration and avidity, it is not surprising that the same trends as S-IgG concentrations were observed. We are also not aware of any studies that have quantified ADCP scores after two doses of COVID-19 vaccines beyond one-month post-vaccination. For context, a study that quantified ADCP scores up to 149 days post-SARS-CoV-2 infection found that as time increased post-COVID-19 infection, ADCP scores decreased^14^. The positive correlation between S-IgG and functional assays (ADCP and ACE2 inhibition) at one-month and four-months post-second dose for mRNA/mRNA and ChAdOx1-S/mRNA supports the hypothesis that the decrease in antibody function by four-months post-second dose is due to decreased S-antibody concentrations rather than the functional capacities of the antibodies^15,16^. Collectively, our results agree with literature findings which suggest that the decrease in S-antibody responses approximately four to six months post-second dose of a COVID-19 vaccine series is due to the natural waning of the immune response post-vaccination.

The observation that mRNA/mRNA and ChAdOx1-S/mRNA recipients responded similarly and had higher responses than ChAdOx1-S/ChAdOx1-S is consistent with literature. It has been reported that S-IgG concentrations were lowest amongst adults vaccinated with ChAdOx1-S/ChAdOx1-S compared with mRNA/mRNA (homologous mRNA-1273 or BNT162b2) or ChAdOx1-S/mRNA vaccinated participants^17^. Similarly, no significant differences in ACE2 neutralization between participants vaccinated with ChAdOx1-S/BNT162b2 and BNT162b2/BNT162b2, but both groups had higher percent inhibition than ChAdOx1-S/ChAdOx1-S participants^18^. The ENFORCE study found ACE2 inhibiting antibody concentrations were highest for ChAdOx1-S/mRNA-1273 and ChAdOx1-S/BNT162b2 compared to homologous mRNA series^13^. However, the participants in the ChAdOx1-S/mRNA group primarily consisted of younger female participants^13^. Kaplonek et al. ^19^ reported that homologous mRNA series resulted in higher S-IgG titres and ADCP scores compared to homologous viral vector series. The lower ADCP scores and ACE2 inhibiting antibody concentrations for our study’s ChAdOx1-S/ChAdOx1-S group are likely due to the lower S-IgG concentrations than mRNA/mRNA and ChAdOx1-S/mRNA recipients.

In this study TRAI was the only humoral immune response measure that did not significantly differ between one- and four-months post-second dose, and by four-months post-second dose was similar between groups. Avidity is dependent on affinity of the binding complex, valence of the antibody and the interaction between the antibody-antigen complex based structural conformation^20^. Avidity increases over time as a result of affinity maturation^21,22^ and has been found to increase over time post COVID-19 vaccination and post-SARS-CoV-2 infection^20^. Because all groups had similar TRAIs by four-months post-second dose, this could suggest that both mRNA and viral vector-based vaccines have similar capacities to activate helper T cells and B cells causing germinal center formation, resulting in somatic hypermutation of immunoglobulin genes and clonal selection of high affinity antibodies. The results from this study suggest that COVID-19 vaccine series consisting of two doses of mRNA vaccines, or a heterologous series of ChAdOx1-S followed by an mRNA vaccine were more immunogenic than homologous ChAdOx1-S series. Antibody avidity reported by Hillus et al.^18^ of S1-IgG found that ChAdOx1-S followed by BNT162b2 resulted in higher avidity antibodies (relative avidity index %) compared with homologous series of BNT162b2 or ChAdOx1-S. This difference in antibody avidity was thought to be due to the extended interval (10–12 vs. 3 weeks) between doses in the ChAdOx1-S/BNT162b2 group, resulting in increased antibody affinity^18^. The shorter recommended interval for the homologous groups may also explain why both groups in the mentioned study had similar S1-IgG avidity^18^. However, our regression analyses did not find a relationship between vaccine interval and avidity, likely due to all participants having extended intervals.

Here we confirmed that COVID-19 mRNA vaccines generate robust S-specific CD4^+^ and CD8^+^ T-cell responses that can be detected up to four-months post-second dose^23^, and that the CD4^+^ T-cell response is larger in magnitude than the CD8^+^ T-cell response. At one-month post-second dose, complete S-specific CD8^+^ T cells positively correlated with ACE2 inhibiting antibody concentrations against the index virus and seven viral variants. The role of CD8^+^ T cells in antibody development following mRNA vaccination is not well understood. However, CXCR5^+^ CD8^+^ T cells have been shown to shape antibody responses in mouse models of influenza vaccination^24^. Regression analyses found positive associations of CD8^+^ T cells with ACE2 inhibiting antibody concentrations at four-months post-second dose. However, it is important to acknowledge that although this association is statistically significant, both responses at four-months post-second dose of CD8^+^ T cells and ACE2 inhibiting antibody concentrations are reduced which may result in a stronger association.

Consistent with literature findings, our study demonstrated that previous SARS-CoV-2 infection resulted in higher antibody responses. Adults previously infected had higher S-RBD concentrations, ACE2 binding inhibiting activity and antibody avidity index after one COVID-19 vaccine compared to SARS-CoV-2 naïve participants post-vaccination with homologous series of mRNA-1273, BNT162b2, or ChAdOx1-S^25^. Participants with a history of SARS-CoV-2 infection (pre-vaccination or after first dose) vaccinated with mRNA/mRNA series had higher ADCP scores (targeting S-RBD) than infection-naïve participants suggesting natural infection significantly increases responses to vaccination^26^. It is difficult to interpret why our previously infected ChAdOx1-S/ChAdOx1-S participants did not have significantly higher responses than infection-naïve participants as demonstrated by mRNA/mRNA participants. It should be considered that this group has a small number of participants; seven infection-naïve and four previously infected. Information was not available for the severity of COVID-19 and time between infection and vaccination. This may suggest that ChAdOx1-S has a lower capacity to activate pre-existing immunological memory specific to SARS-CoV-2 in older adults. Gluck et al.^25^ found higher responses in previously infected adults vaccinated with one COVID-19 dose than infection-naïve that received either homologous mRNA-1273, BNT162b2 or ChAdOx1-S series. However, the median age of their cohort was ~ 40 years old and no differences were reported based on vaccine(s) recieved^25^.

Multivariable regression models found that two doses of ChAdOx1-S, no previous history of SARS-CoV-2 infection, lower BMI (healthy or underweight), increased vaccine interval (≥ 13 weeks), and excellent health status were associated with lower humoral immune responses. Two doses of ChAdOx1-S were associated with decreased humoral immune responses in comparison to two doses of mRNA vaccines or ChAdOx1-S followed by one dose of mRNA vaccines indicating they are less immunogenic, resulting in lower responses^17^. Levins et al.^27^ (2021) found a BMI above 30 was associated with higher neutralizing antibody titres post-BNT162b2 vaccination. It was also reported that peak neutralizing antibody levels post-SARS-CoV-2 infection were positively related with obesity^28^. The reason behind this observation is unclear. Excellent health status was associated with lower humoral immune responses which is opposite of what was expected. However, higher BMI (overweight and obese) was associated with higher antibody responses which may suggest why individuals with less than excellent health had higher responses as overweight and obese BMI is associated with poorer lifestyle factors^29^. The negative relationship between antibody response and vaccine interval in this study is contrary to current literature^30–32^. However, it is important to consider that none of our participants had an interval lower than seven weeks. In a study by Skowronski et al. ^4^ based on vaccine effectiveness data from British Columbia and Quebec, it was reported that a minimum of seven to eight week interval may be the optimal compared with shorter intervals for adults 18 years old and older. Seven weeks was the lowest interval in our study therefore we were unable to compare responses to shorter intervals.

To determine the proportions of participants protected against symptomatic infection, GMCs of S-IgG concentrations were compared to suggested literature COVID-19 CoP^11,12^. Regardless of which CoP was used, nearly all mRNA/mRNA and mRNA/ChAdOx1-S participants were considered protected, but a maximum proportion of 63.6% of ChAdOx1-S/ChAdOx1-S were considered protected using the lowest CoP of 60 BAU/mL. This was expected as mRNA/mRNA and mRNA/ChAdOx1-S had very similar S-IgG concentrations post-second dose, which were higher than ChAdOx1-S/ChAdOx1-S responses. It is also important to note that although S-IgG concentration decreased between timepoints, TRAI antibody avidity increased over time and did not differ between vaccine series received. Our data collectively suggests that antibody concentration (S-IgG) alone may not indicate protection against SARS-CoV-2 infection.

To determine the relationship between S-IgG concentration and avidity, multivariable linear regression analyses were performed using FAA to determine if antibody concentration or specific avidity level(s) had a larger role in S-specific antibody function. For both ADCP scores and concentrations of ACE2 inhibiting antibodies specific for index spike, all FAA levels were statistically significant at one-month post-second dose. This suggests that both S-IgG concentration and avidity are equally important for function, indicating that a higher concentration of low avidity antibodies or lower concentration of high avidity antibodies will result in a similar level of function. Based on this observation, the correlation between TAA and ADCP scores and ACE2 inhibiting antibody concentrations was also performed to identify S-antibody functions that could also be used along with S-IgG concentration as a CoP. Both ADCP scores and ACE2 inhibiting antibody concentrations positively correlated with TAA for mRNA/mRNA and mRNA/ChAdOx1-S groups but not ChAdOx1-S/ChAdOx1-S. Our data suggests that S-IgG concentration and avidity are important determinants of antibody function.

Our cohort consisted of community-dwelling older adults that received both homologous and heterologous vaccine series from individuals that had extended vaccine intervals which accurately represent the heterogeneity of the COVID-19 vaccine roll out in Canada. Limitations of our study include the overall number of participants and lack of participants recruited at earlier visits (baseline to pre-second dose) due to rapid vaccine rollout in this age group, which provided challenges for enrollment. We also could not perform paired statistical analyses between study timepoints due to participants joining the study at different timepoints and missed study visits. This resulted in participants being grouped based on vaccine type (mRNA vs. viral vector) rather than based on specific vaccine product(s) received. An additional consequence of the smaller cohort size is that these statistical analyses are underpowered resulting in a greater possibility of type II error. There was also a lack of diversity in our group of participants with respect to sex, gender and ethnicity. T-cell analyses were unable to be performed on individuals vaccinated with ChadOx1-S/ChadOx1-S, which would have provided meaningful comparisons between groups. For ACE2 inhibiting antibody concentrations, results are often reported as percent inhibition, but due to high variation in antibody concentrations, samples were analyzed at two different dilutions which could not be accounted for with percent inhibition. A limitation of the ACE2 surrogate neutralization assay is that it only measures the ability of anti-S antibodies to block the interaction of ACE2 receptor and the RBD of the S-protein. This assay only measures antibodies that prevent binding by direct competition of the binding site and is not able to quantify other neutralizing abilities of antibodies outside of inhibiting entry into cells.

## Conclusions

This study provides important data on S-specific antibody concentration and function as well as T-cell responses in community-dwelling older adults. All three vaccines were immunogenic, but greater responses were seen in participants with a series containing least one mRNA vaccine. Antibody avidity should be considered as a potential CoP because it quantifies not only amounts of antigen-specific antibodies but also the binding strength of these antibodies. Because all FAA had a strong positive correlation with antibody function, a measure that takes into consideration both concentration and function should be considered when determining CoP. The next step of this study is to continue quantifying responses to booster doses in older community-dwelling adults.

## Methods

### Participant recruitment and sample collection

This study was approved by the University of British Columbia Research Ethics board (REB number: H20-03951) and was performed in accordance with the Declaration of Helsinki. Responses to COVID-19 vaccination were quantified in participants enrolled to the ‘PRospEctiVe EvaluatioN of immuniTy after COVID-19 vaccines’ (PREVENT-COVID) study over five study visits. Participants were recruited via sending study information through local networks; advertising through public media, existing volunteer or participant databases, institute-related websites and social media outlets; posters in physician offices, local hospitals, university areas and other COVID-19 immunization sites. Inclusion criteria were: ≥ 19 years old at first visit; and had an immunization provider administer a COVID-19 vaccine. All participants provided written informed consent. Exclusion criteria consisted of bleeding disorders that contraindicated blood collection, immunocompromising conditions or currently taking medication that may affect immune responses to COVID-19 vaccination. This analysis only included participants aged ≥ 50 years.

Using early published binding antibody data from the mRNA-1273 vaccine^33^, 50 individuals per group was determined to be sufficient to establish superiority between groups with 0.95 power (0.05 level of significance) and allowing for 10% loss to follow-up at each study visit. Data collected included age, sex, gender, ethnicity, health status (excellent, very good, good, fair, mildly poor, poor, very poor) ^34–36^ (Supplementary table 10), height, weight, COVID-19 vaccine product, dates of vaccinations, health conditions, and SARS-CoV-2 PCR testing data. Participants attended a maximum of five visit timepoints from April to December 2021. Participants could enter the study at any time from prior to their first vaccine dose to one-month after their second dose. Pre-vaccination timepoints were eligible for collection up to 24 h pre-vaccination. One-month post-dose vaccination were within window for collection between three- and six-weeks post vaccination. The last sample (four-months post-second dose) was eligible for collection between three- and five-months post-second dose. Participants were separated based on vaccine series as well as previous history of SARS-CoV-2 infection (Fig. 1). Participant groups were based on vaccine type received (mRNA or viral vector vaccines) due to small cohort size. The mRNA/mRNA group consisted of either homologous or heterologous series mRNA-1273 or BNT162b2 and the ChAdOx1-S/mRNA group received ChAdOx1-S followed by either mRNA-1273 or BNT162b2. There was a total of four participant samples that were collected out of the one-month post-second dose window by approximately one week. A sensitivity analysis was performed, and determined that there was no significant difference in results when these participants were removed, therefore they remained in the final study analyses.

### Determination of COVID-19 infection status

To ensure that responses to vaccination were not due to potential SARS-CoV-2 infection, participant provincial health records were screened for any positive COVID-19 PCR tests^37^. The following commercial assays were also performed on serum collected at each visit; Siemens ADVIA Centaur SARS-CoV-2 spike protein S1 RBD (total antibody detection), Ortho Vitros SARS-CoV-2 spike S1 RBD (total antibody detection) and Abbott Architect SARS-CoV-2 nucleocapsid (total IgG detection) to identify SARS-CoV-2 previously infected participants. For pre-vaccination samples, participants were identified as SARS-CoV-2 previously infected if any of the commercial assays had a reactive result. At post-vaccination visits, samples had to be reactive for both the nucleocapsid (Abbott) assay and either spike protein S1 RBD (Siemens or Ortho Vitros) assays to be considered previously infected^38,39^. Once a participant was identified as SARS-CoV-2 previously infected, they were considered previously infected for subsequent study visits.

### Enzyme-linked immunosorbent assay (ELISA)

An indirect ELISA was performed for all available serum samples. Ninety-six well flat bottom microtiter plates (Immulon 2 HB, Thermo Fisher Scientific) were coated with SARS-CoV-2 index virus spike protein (SMT1-1 SARS-CoV-2 spike glycoprotein reference material, National Research Council Canada). Serum and standard (20/136, National Institute for Biological Standards and Control) were diluted in antibody buffer (1% skim milk in 1X PBS- 0.1% Tween20, Thermo Fisher Scientific). Plates were washed with 1X-PBS-0.1% Tween20 (wash buffer) before the addition of the secondary antibody (mouse anti-human IgG, Fc fragment specific (HP6043) peroxidase conjugate, Millipore). The substrate solution was added (O-Phenylenediamine dihydrochloride (OPD), Sigma Aldrich) prior to the addition of 3 M hydrochloric acid (Thermo Fisher Scientific). Optical densities of individual wells were analyzed at 490 nm (infiniteM200, Tecan). Concentrations of anti-spike protein specific IgG (S-IgG), expressed in binding antibody units per millilitre (BAU/mL). Samples below 2 BAU/mL were assigned a value of 1 BAU/mL for statistical purposes.

### Antibody avidity assay

Using the ELISA described above, 1X PBS, 0.25 M, 0.5 M, 0.75 M, 1.0 M, 1.5 M and 2.0 M ammonium thiocyanate (NH_4_SCN, Fisher Scientific) was added to seven separate wells prior to the addition of the secondary antibody, similarly to Abu-Raya et al.^40^. The results were reported as the total relative avidity index (TRAI) expressed in arbitrary units (AU) and the total absolute avidity levels (TAA) expressed in absolute AU per mL (AAU/ mL). The minimum concentration needed to perform the assay was not reached for any of the baseline samples (20 BAU/mL), therefore no comparisons could be made between baseline and subsequent visits. Fractional absolute avidity levels (FAA) of S-IgG present at each concentration of NH_4_SCN were reported in BAU/mL. Supplementary Fig. 5 displays a sample calculation.

### Antibody dependent cellular phagocytosis (ADCP) assay

This assay was optimized based on previously published material^41–44^. Briefly, fluorescent beads (FluoSpheres™ NeutrAdavin ™, 1 µm, yellow-green, 505/515, Invitrogen) were conjugated with biotinylated SARS-CoV-2 index virus spike protein (Biotinylated SARS-CoV-2 S protein, His,Avitag™, Super stable trimer, MALS verified, ACROBiosystems) before being added to participant serum. THP-1 cells (American Type Culture Collection) were added to each well with the bead-antibody complexes overnight to allow the cells to phagocytose the beads. Samples were analyzed in triplicate with the BD LSR Fortessa flow cytometer and FlowJo Software (BD Biosciences). The gating strategy is displayed in Supplementary Fig. 6. Results were reported as the mean phagocytic score of duplicates resulting the lowest percent coefficient of variation (CV) (percentage of bead positive events multiplied by the mean fluorescent intensity of the bead positive events). Percent CV of duplicates had to be below 20% to be accepted. Results below a mean phagocytic score of 300 were assigned a score of 150 for statistical purposes.

### Angiotensin-converting enzyme 2 (ACE2) inhibition assay

Serum samples were diluted in Meso Scale Discovery (MSD) Diluent 100 prior to serological testing. Following the manufacturer recommended protocol, we measured the capacity of spike specific SARS-CoV-2 antibodies directed against the index virus and nine additional variants (WHO label^45^, pango lineage^46^) index virus (GISAID Accession ID: EPI_ISL_402124), Alpha (B.1.1.7), Beta (B.1.351), Gamma (P.1), Delta (B.1.617.2), Zeta (P.2), Iota (B.1.526.1), Kappa (B.1.617.1), B.1.617, and B.1.617.3 to inhibit the interaction of the ACE2 receptor with the spike protein using the MSD V-PLEX SARS-CoV-2 Panel 13 ACE2 assay. Inhibition capacity was measured using the MSD QuickPlex SQ120 instrument and was reported as concentration of ACE2 inhibiting antibodies, in μg/mL for the index virus and in units/mL for SARS-CoV-2 variants. Results below the following lower limits of quantification were assigned half of the following values: index virus (0.02262 μg/mL), Alpha (0.01620 units/mL), Beta (0.00762 units/mL), Gamma (0.01439 units/mL), Delta (0.03334 units/mL), Zeta (0.01655 units/mL), Iota (0.01761 units/mL), Kappa (0.007091 units/mL), B.1.617 (0.02540 units/mL), B.1.617.3 (0.01044 units/mL).

### Activation induced marker (AIM) assay

Whole blood was collected in sodium heparin tubes, kept at room temperature and processed within 4 h of collection. Blood was mixed at 1:1 ratio with IMDM media (Gibco), and 200 µL of whole blood-media mixture was added to a single well per condition in a 48-well tissue culture plate. Wells were stimulated with 1 μg/mL of SARS-CoV-2 (index virus) overlapping peptide pool of the immunodominant regions of the spike glycoprotein (Prot_S PepTivator®, Miltenyi Biotec), or the complete spike glycoprotein complete (Complete Prot_S PepTivator®, Miltenyi Biotec). Negative control wells were left unstimulated. Assays were incubated at 37 °C for 44–48 h. Anti-CD137-APC mAb (BioLegend, Supplementary table 11) was added to each condition at stimulation, as upregulation of this surface receptor peaks at 24 h. Following stimulation, whole blood was surface stained with mAb panel for 20 min at room temperature. One milliliter of OptiLyse C (Beckman Coulter) was added to each tube and incubated for 10 min at room temperature in the dark. Cells were washed twice with 2 mL of PBS and fixed with 0.5% paraformaldehyde in PBS and stored at 4 °C until acquisition. Samples were acquired within 48 h of staining on a 5-laser A5 Symphony cytometer (BD Biosciences), fresh single colour controls were used for compensation. Phenotypic analysis was performed using FlowJo version 10.8.1 (BD Biosciences). Assay cut-off for a positive response (CD4^+^CD25^+^OX40^+^ or CD8^+^CD69^+^4-1BB^+^) was 0.02%, being the mean + 3SD of the negative control wells.

### Statistical analyses- humoral immune responses

To evaluate humoral immune responses to COVID-19 vaccines in adults over 50 years old, S-antibody concentration and function were quantified. Participants were separated into three groups based on the types of first and second COVID-19 vaccine doses received. The first group received either two doses of BNT162B2 or two doses of mRNA-1273 or a combination of BNT162B2 and mRNA-1273 (mRNA/mRNA), the second group received ChAdOx1-S for their first dose and either BNT162B2 or mRNA-1273 as their second dose (ChAdOx1-S/mRNA), and the third group received two doses of ChAdOx1-S (ChAdOx1-S/ChAdOx1-S).

To assess the suggested protection elicited by a two-dose series of COVID-19 vaccines, S-IgG concentrations were compared to suggested literature CoP. Goldblatt et al.^11^ suggested CoP against symptomatic infection corresponding to 154 BAU/mL based on homologous mRNA (mRNA-1273 or BNT162b2) or viral vector (ChAdOx1-S or Ad.26COV2.S). They also removed mRNA vaccine responses from their analyses which resulted in a correlate of 60 BAU/mL (95% CI 35–102) because it may be useful when comparing vaccines with lower vaccine efficacy. Wei et al. ^12^ suggested potential correlates of protection based on S-IgG concentrations by estimating two-thirds (67%) of individuals were protected against infection. Homologous ChAdOx1-S or homologous BNT162b2 required estimated concentrations of 107 BAU/mL or 94 BAU/mL. Using models that pooled data for both vaccines, 100 BAU/mL was suggested^12^.

Prior to statistical analyses, all data were log_10_ transformed. Statistical analyses were performed with R (V.4.1.1; The R Project for Statistical Computing, Vienna, Austria). R version 4.1.3 (2022-03-10). Statistical analyses were performed on groups with a minimum of two participants. Results were considered statistically significant at *P*-values < 0.05. A Welch’s t-test was used to compare responses to vaccination based on S-IgG concentrations, antibody avidity, ADCP scores and ACE2 inhibiting antibody concentrations between timepoints for each group, as well as compare previously infected and infection-naïve participants within the same group at each study timepoint. For these comparisons, a Bonferroni correction was applied adjusting the *P*-values by multiplying by the number of comparisons (seven). A One-way ANOVA was used to compare the three groups at each visit (Tukey–Kramer post-hoc). Multivariable linear regression analyses were performed to determine the effect of demographic factors on S-IgG concentrations, TRAI, ADCP scores and ACE2 inhibiting antibody concentrations for the index virus. Based on the distribution of the data, demographic factors were separated into the following groups: age (≥ 70 years old vs. ≤ 69 years old), sex (male vs. female), ethnicity (white vs. non-white), BMI (normal (18.5–24.9) and underweight (< 18.5) combined vs. overweight and obese (≥ 25) combined, vaccine series (ChAdOx1-S/ChAdOx1-S vs. mRNA/mRNA and ChAdOx1-S/mRNA combined), vaccine interval (≥ 13 weeks vs. < 13 weeks), health status (excellent vs. very good/ good/ fair/ mildly poor combined) and infection status (previously infected vs. infection-naïve) at one-month post-second dose. This was achieved by fitting the multivariable model via priori-define backwards selection protocol with the Bayesian Information Criterion (BIC) statistic used to select the model with the best fit. From univariable models, the initial multivariable model included all demographic factors with a *P* < 0.10. The reduced models were selected by removing the demographic factor with the highest *P*-value at each stage. The final model consisted of the demographic factors with the lowest BIC value^47^.

To determine the contributions of both antibody concentration and avidity on antibody function, multivariable linear regression analyses were completed for one-month post-second dose between FAA and ADCP scores as well as ACE2 inhibiting antibody concentrations. Multivariable models were fit using a conservative confounding model selection approach. From univariable models, the initial multivariable model included all demographic factors with a *P* < 0.10. A stepwise approach was applied to fit a series of reduced models. The value of the coefficient was compared between reduced model for each FAA, the secondary demographic factor associated with the lowest relative change were removed. This process was repeated until the minimum change exceeded 5%^48^.

Spearman correlations were performed to determine the relationship between S-IgG concentrations and antibody function (TRAI, ADCP scores and concentrations of index virus ACE2 inhibiting antibodies) at one-month and four-months post-second dose. The same approach was taken to evaluate the correlation between TAA and antibody function (ADCP scores and concentrations of index virus ACE2 inhibiting antibodies). A Bonferroni correction was applied adjusting the *P*-values by multiplying by the number of tests performed (eight).

### Statistical analyses (T-cell responses)

All statistical analyses were performed in R studio (version 4.0.2 and below). T cell data were tested for normal distribution and equal variance using Shapiro–Wilk (*shapiro.test()*) and Levene Test (*leveneTest()*) respectively. Kruskal Wallis (*kruskal.test()*) and ANOVA(*anov()*) were performed based on distribution and variance for multiple comparisons. If significance was found (*P* < 0.05), Wilcoxon Rank Sum Test (*pairwise.wilcox.test()*) or Tukey’s Multiple Comparisons test (*glth() using mcp* = *“TUKEY”*) were carried out. Mann–Whitney (*wilcox.test()*) tests were used when comparing only two groups. Multilinear regression models were generated to determine the impact of demographic data on T-cell responses and T-cell responses on humoral immune responses. The associations between T cell data from one-month post-second dose and four-months post-second dose and humoral immune responses at the same time point were evaluated. Additionally, the associations between T cell data from one-month post-second dose and humoral immune responses from four-months post-second dose were evaluated. Heatmaps were generated and scaled using lm() function.

### Supplementary Information

Supplementary Information.

## Data Availability

The datasets generated and/or analysed during the current study are not publicly available as the study is ongoing but are available from the corresponding author on reasonable request.
