# Supplementary material for: Adaptive immune responses to two-dose COVID-19 vaccine series in healthy Canadian adults ≥ 50 years: a prospective, observational cohort study
Source: Sci Rep. 2024 Apr 18;14:8926. doi: 10.1038/s41598-024-59535-0 (PMC11026432; doi:10.1038/s41598-024-59535-0)
Supplement: Supplementary file 1 — Supplementary Information. [file 41598_2024_59535_MOESM1_ESM.docx]

Adaptive immune responses to two-dose COVID-19 vaccine series in healthy Canadian adults ≥ 50 years: a prospective, observational cohort study

Gabrielle N Gaultier^1,2*^, Brynn McMillan^3,2^, Chad Poloni^4,5^, Mandy Lo^1,2^, Bing Cai^1,2^, Jean J Zheng^4,2^, Hannah M Baer^4,5,6^, Hennady P Shulha^1,2^, Karen Simmons^1,2^, Ana Citlali Márquez^7^, Sofia R Bartlett^7,8^, Laura Cook^5,9,10^, Megan K Levings^5,11,12^, Theodore Steiner^5,9^, Inna Sekirov^7,13^, James E A Zlosnik^7^, Muhammad Morshed^7,13^, Danuta M Skowronski^7,8^, Mel Krajden^7,13^, Agatha N Jassem^7,13^, Manish Sadarangani^1,2^

^1^Department of Pediatrics, University of British Columbia, Vancouver, British Columbia, Canada

^2^Vaccine Evaluation Center, British Columbia Children’s Hospital Research Institute, Vancouver, British Columbia, Canada

^3^Experimental Medicine Program, University of British Columbia, Vancouver, British Columbia, Canada

^4^Department of Microbiology and Immunology, University of British Columbia, Vancouver, British Columbia, Canada

^5^British Columbia Children’s Hospital Research Institute, University of British Columbia, Vancouver, British Columbia, Canada

^6^Institute of Infection, Inflammation & Immunity, College of Medical, Veterinary and Life Sciences, University of Glasgow, Glasgow, UK.
^7^British Columbia Centre for Disease Control, Vancouver, British Columbia, Canada
^8^School of Population and Public Health, University of British Columbia, Vancouver, British Columbia, Canada

^9^Department of Medicine, University of British Columbia, Vancouver, British Columbia, Canada

^10^Department of Microbiology & Immunology, University of Melbourne at the Peter Doherty Institute for Infection and Immunity, Melbourne, Victoria, Australia
^11^Department of Surgery, University of British Columbia, Vancouver, British Columbia, Canada
^12^School of Biomedical Engineering, University of British Columbia, Vancouver, British Columbia, Canada
^13^Department of Pathology and Laboratory Medicine, University of British Columbia, Vancouver, British Columbia, Canada


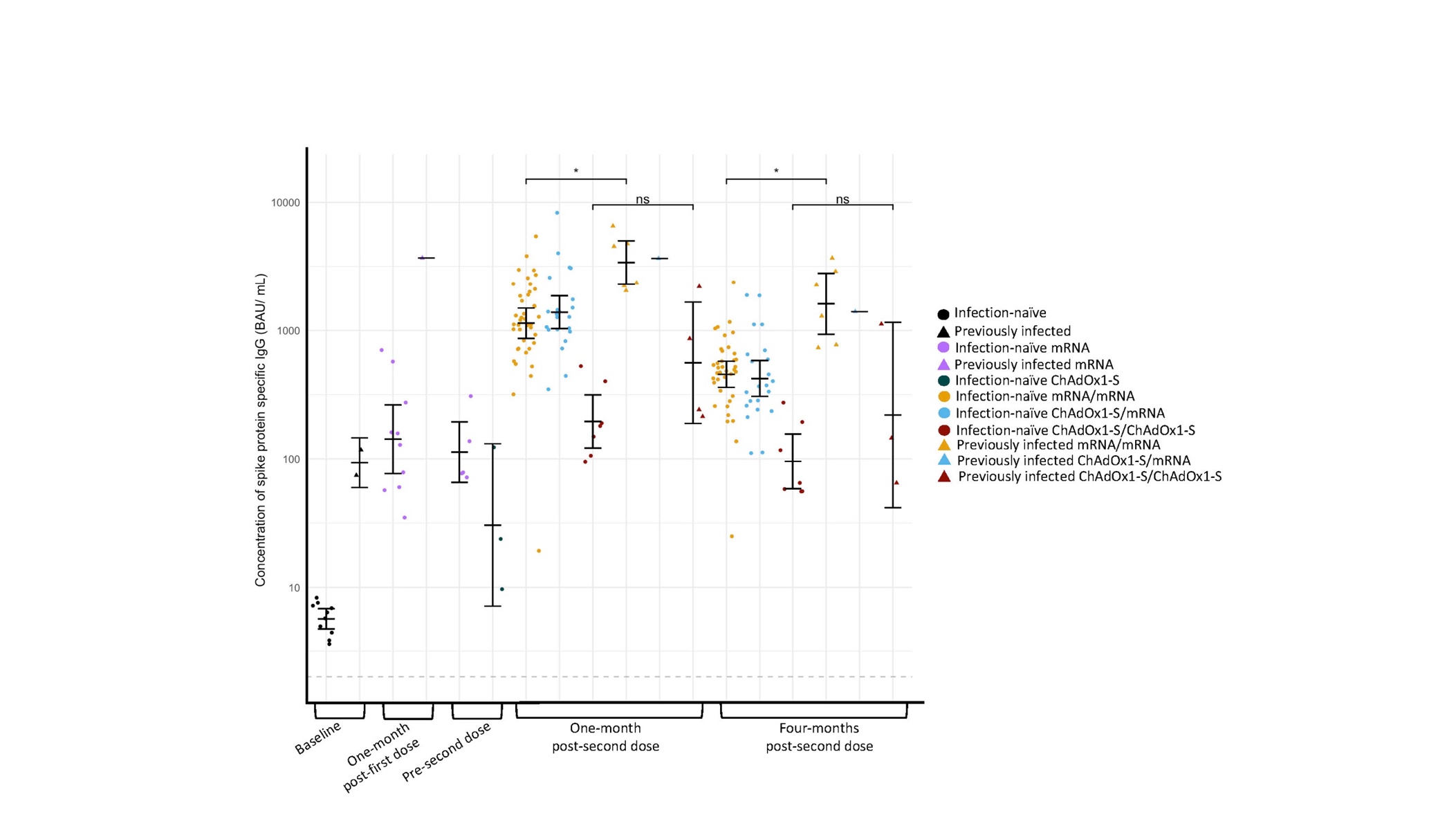


**Supplementary fig 1**. **Geometric mean concentrations (GMC) of anti-spike protein IgG in binding antibody units per milliliter (BAU/ mL) of infection-naïve and previously infected participants grouped based on vaccine series**. The GMC is represented by the solid line with 95% confidence intervals. Data were log_10_ transformed prior to statistical analyses. The grey dashed line represents the lower limit of quantification (LLQ) of 2 BAU/ mL, values below the LLQ were assigned a value of 1 BAU/ mL for statistical purposes.

**P*<0.05, compared concentrations of anti-spike protein IgG between infection-naïve and previously infected participants that received the same vaccine series at study visits (Welch’s t-test, a Bonferroni correction was applied adjusting the P-values by multiplying by the number of comparisons (seven)). Not significant (ns) *P*>0.05. Baseline (infection-naïve n=10) (previously infected n=2), one-month post-first dose (infection-naïve mRNA n=10) (previously infected mRNA n=1), pre-second dose (infection-naïve mRNA n=5) (infection-naïve ChAdOx1-S n=3), one-month post-second dose (infection-naïve mRNA/mRNA n=41) (infection-naïve ChAdOx1-S/mRNA n=22) (infection-naïve ChAdOx1-S/ChAdOx1-S n=7) (previously infected mRNA/mRNA n=6) (previously infected ChAdOx1-S/mRNA n=1) (previously infected ChAdOx1-S/ChAdOx1-S n=4), four-months post-second dose (infection-naïve mRNA/mRNA n=38) (infection-naïve ChAdOx1-S/mRNA n=22) (infection-naïve ChAdOx1-S/ChAdOx1-S n=7) (previously infected mRNA/mRNA n=6) (previously infected ChAdOx1-S/mRNA n=1) (previously infected ChAdOx1-S/ChAdOx1-S n=3).

**
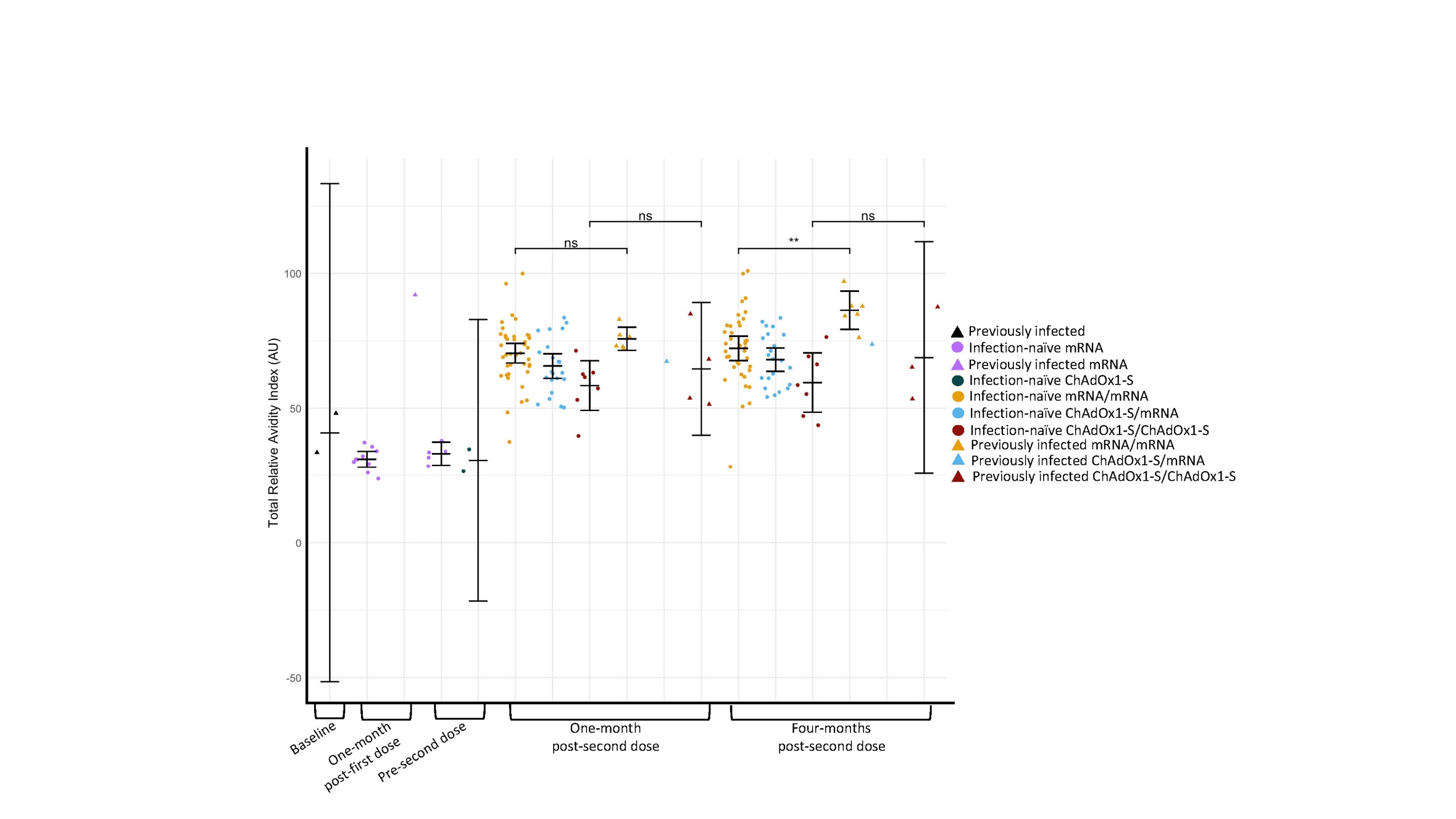
**

**Supplementary fig 2**. **Anti-spike protein specific IgG avidity (total relative avidity index) of infection-naïve and previously infected participants grouped based on vaccine series**. Total Relative Avidity Index (TRAI) of anti-spike protein IgG in arbitrary units (AU); mean TRAI is represented by the solid line with 95% confidence intervals.

**P*<0.05, ***P*<0.01, ****P*<0.001, compared concentrations of anti-spike protein IgG between infection-naïve and previously infected participants that received the same vaccine series at study visits (Welch’s t-test, a Bonferroni correction was applied adjusting the P-values by multiplying by the number of comparisons (seven)). Not significant (ns) P>0.05. Baseline (previously infected n=2), one-month post-first dose (infection-naïve mRNA n=10) (previously infected mRNA n=1), pre-second dose (infection-naïve mRNA n=5) (infection-naïve ChAdOx1-S n=2), one-month post-second dose (infection-naïve mRNA/mRNA n=41) (infection-naïve ChAdOx1-S/mRNA n=22) (infection-naïve ChAdOx1-S/ChAdOx1-S n=7) (previously infected mRNA/mRNA n=6) (previously infected ChAdOx1-S/mRNA n=1) (previously infected ChAdOx1-S/ChAdOx1-S n=4), four-months post-second dose (infection-naïve mRNA/mRNA n=38) (infection-naïve ChAdOx1-S/mRNA n=22) (infection-naïve ChAdOx1-S/ChAdOx1-S n=7) (previously infected mRNA/mRNA n=6) (previously infected ChAdOx1-S/mRNA n=1) (previously infected ChAdOx1-S/ChAdOx1-S n=3).


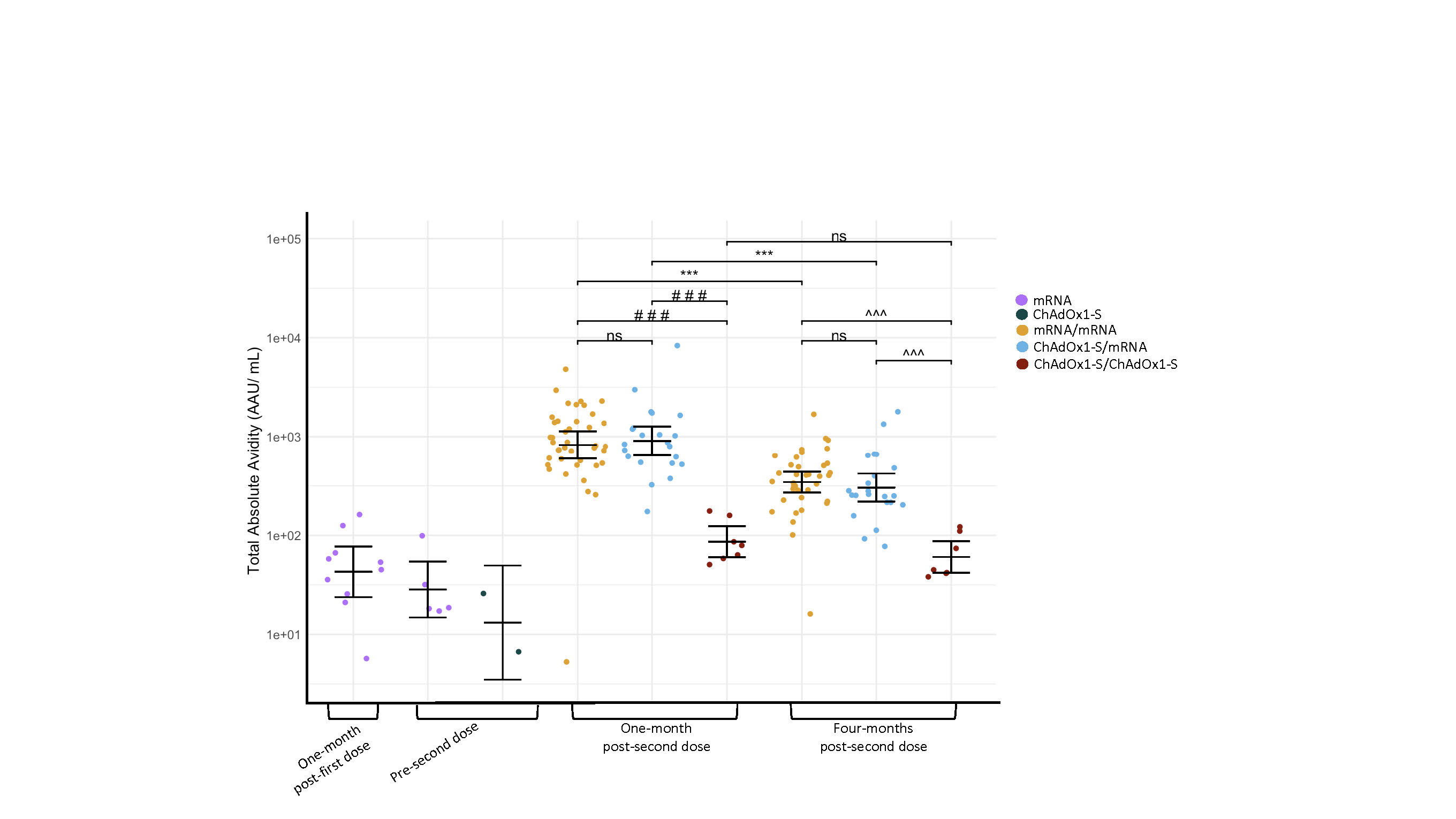


**Supplementary fig 3**. **Anti-spike protein specific IgG avidity (total absolute avidity levels) of infection-naïve participants grouped based on vaccine series**. Total absolute avidity levels (TAA) of spike protein specific IgG in arbitrary units (AAU/ mL); geometric mean TAA is represented by the solid line with 95% confidence intervals. Data were log_10_ transformed prior to statistical analyses.

****P*<0.001, compared concentrations of anti-spike protein IgG between study visits within the same group (Welch’s t-test, a Bonferroni correction was applied adjusting the *P*-values by multiplying by the number of comparisons (seven)). ###*P*<0.001, compared concentrations of anti-spike protein IgG between groups at one-month post-second dose (One-way ANOVA, Tukey-Kramer post-hoc). ^^^*P*< 0.001, compared concentrations of anti-spike protein IgG between groups at four-months post-second dose (One-way ANOVA, Tukey-Kramer post-hoc). Not significant (ns) *P*>0.05. One-month post-first dose (mRNA n=10), pre-second dose (mRNA n=5) (ChAdOx1-S n=2), one-month post-second dose (mRNA/mRNA n=41) (ChAdOx1-S/mRNA n=22) (ChAdOx1-S/ChAdOx1-S n=7), four-months post-second dose (mRNA/mRNA n=38) (ChAdOx1-S/mRNA n=22) (ChAdOx1-S/ChAdOx1-S n=7).


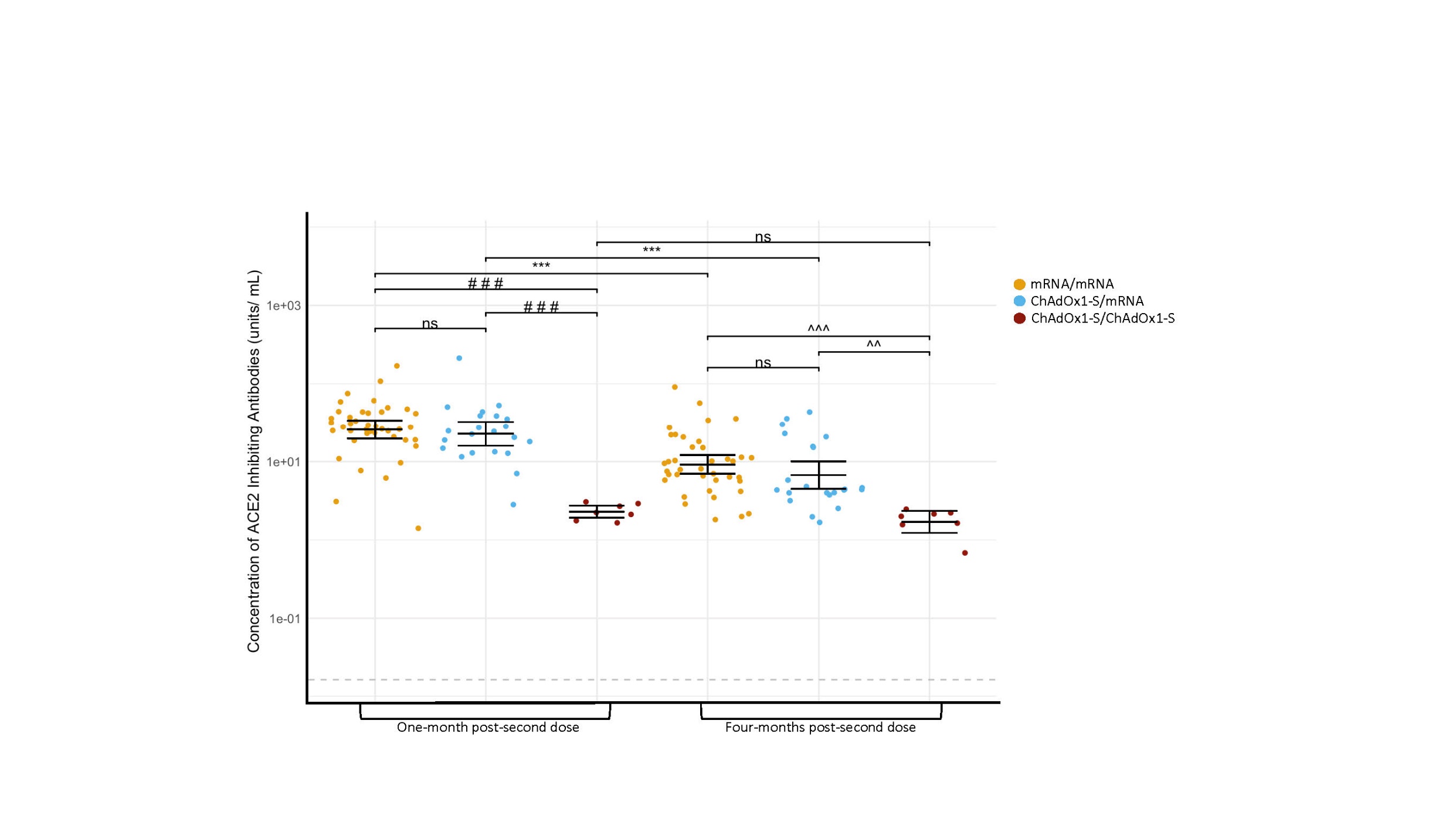


**4a**

**4b**


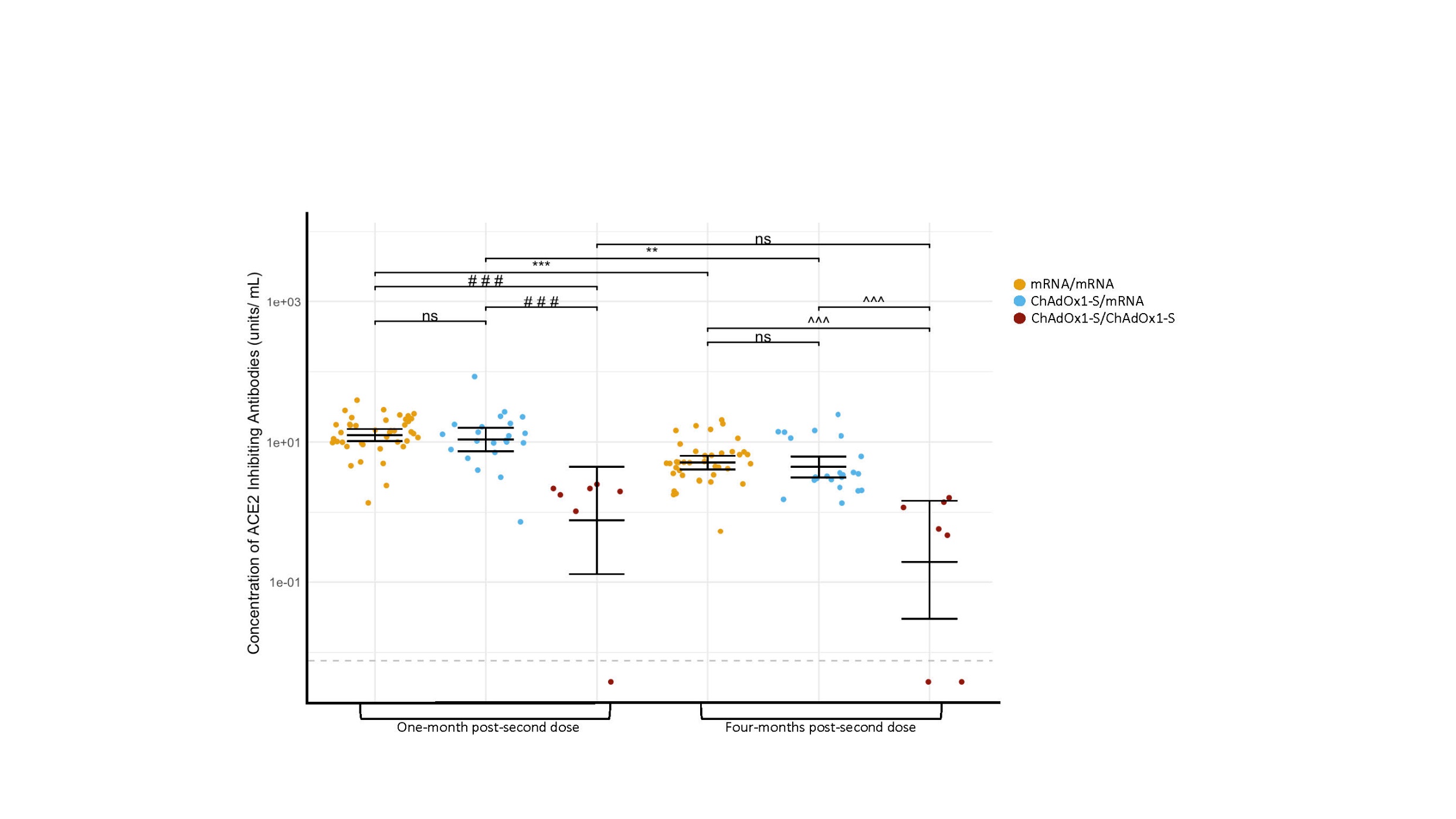


**4c**


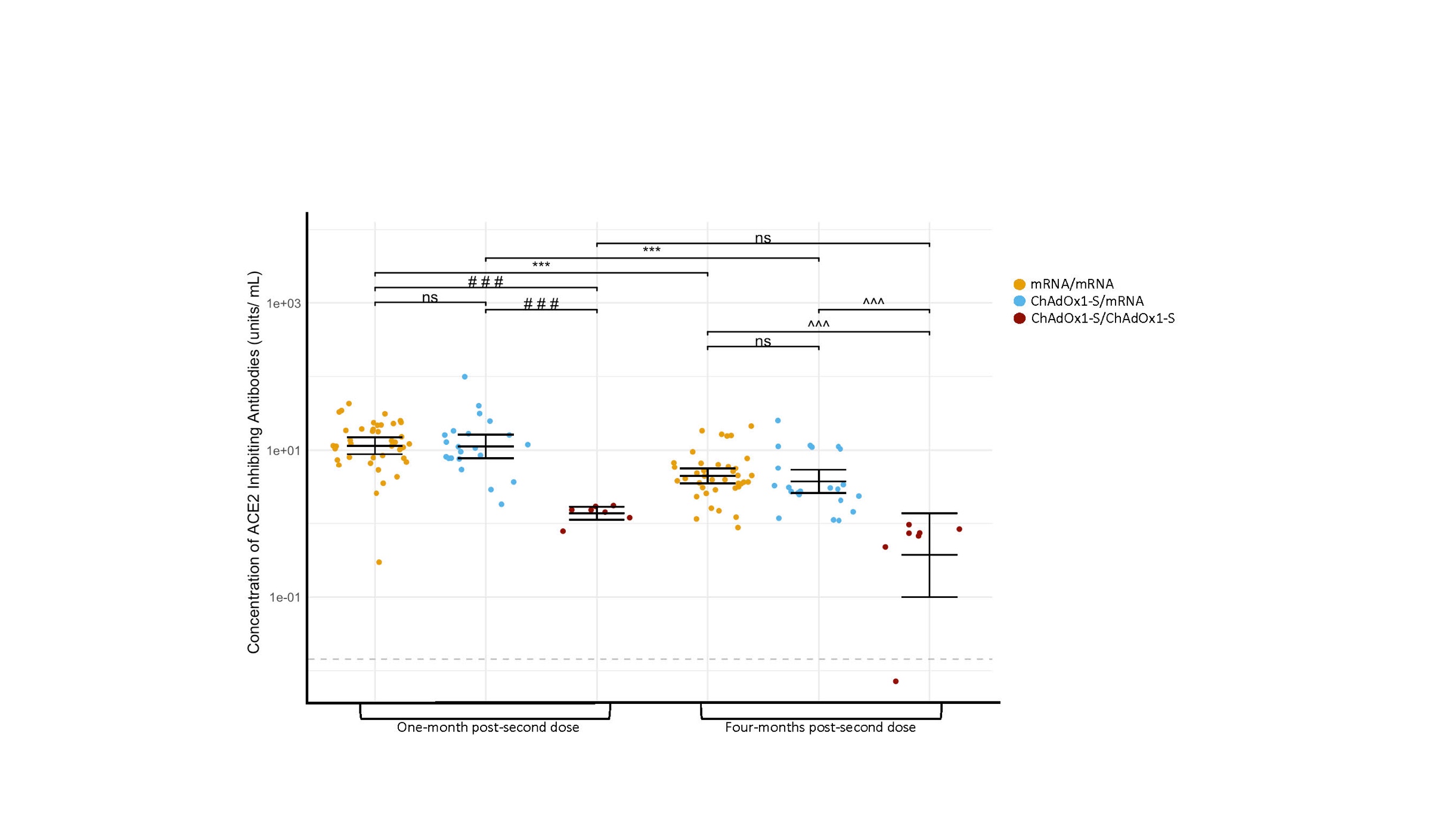


**4d**


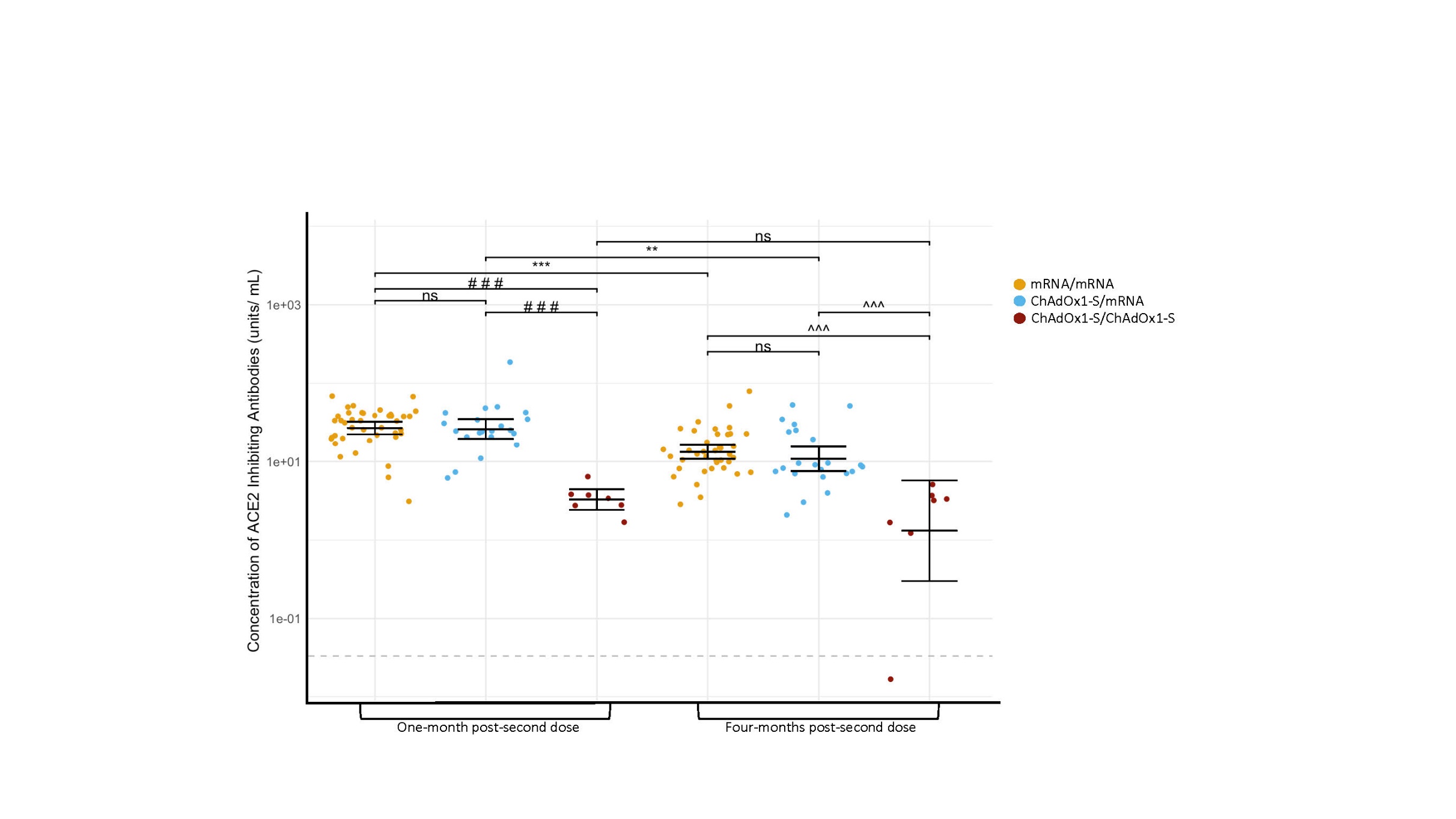


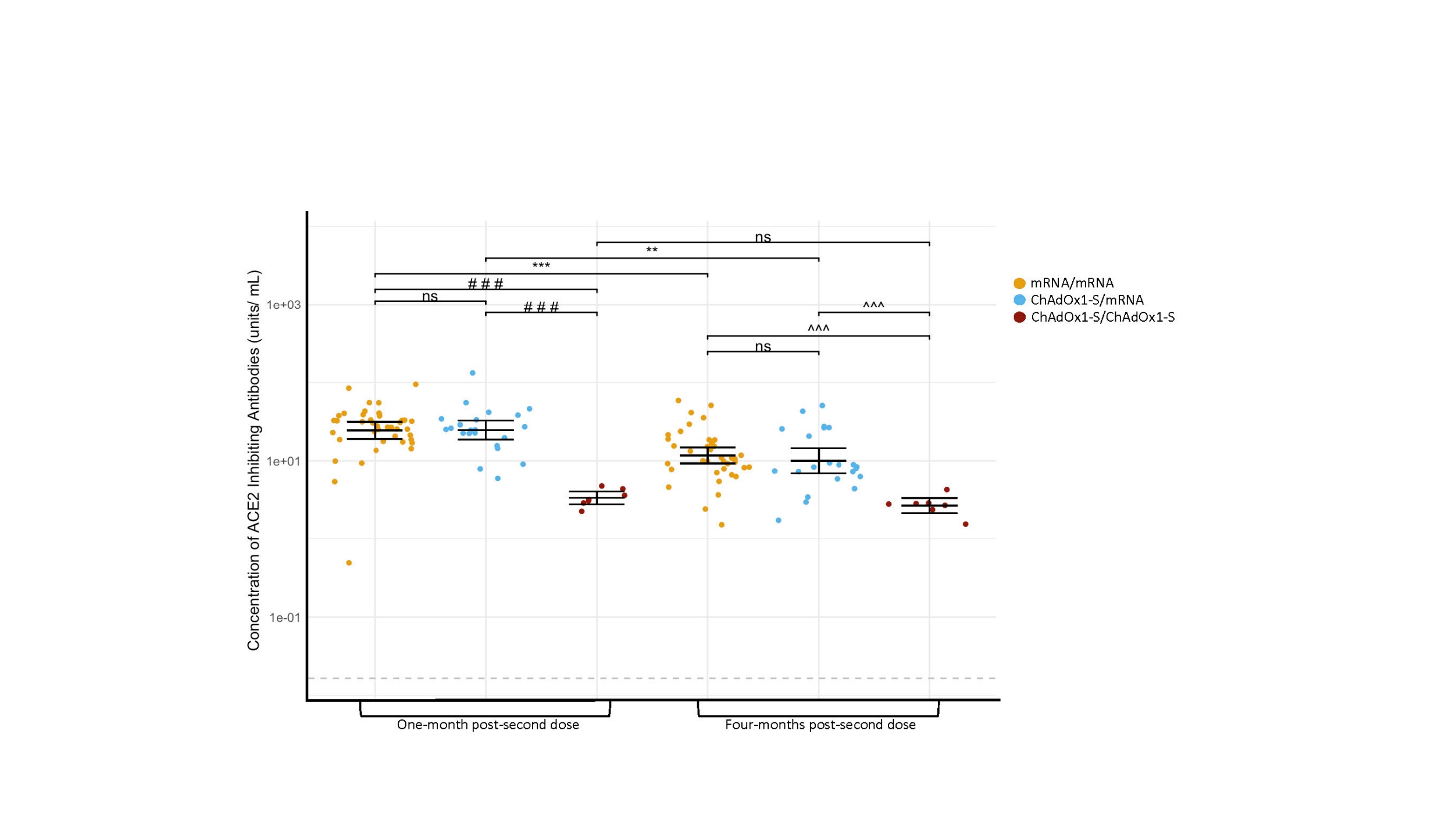


**4e**

**4f**


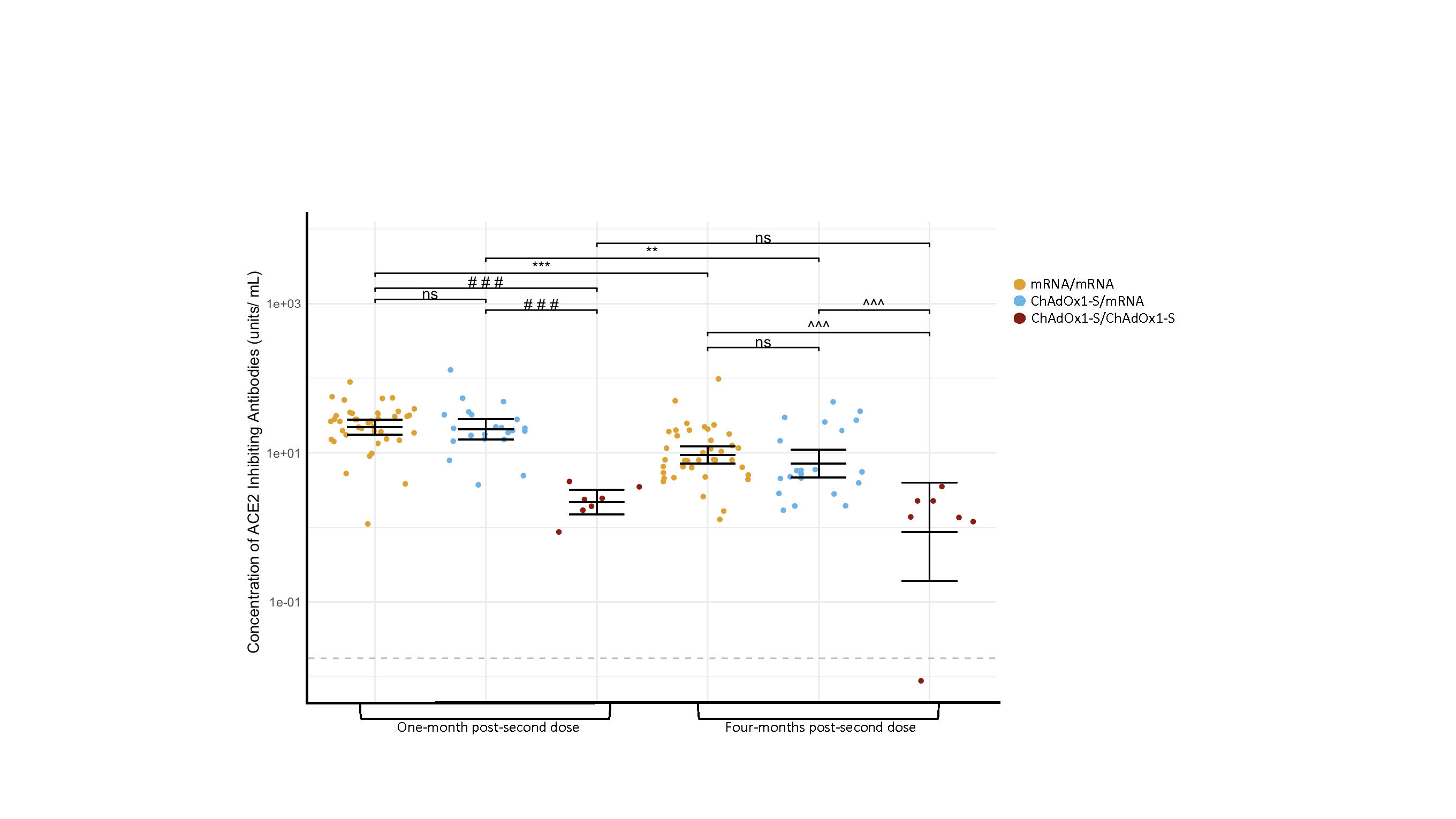


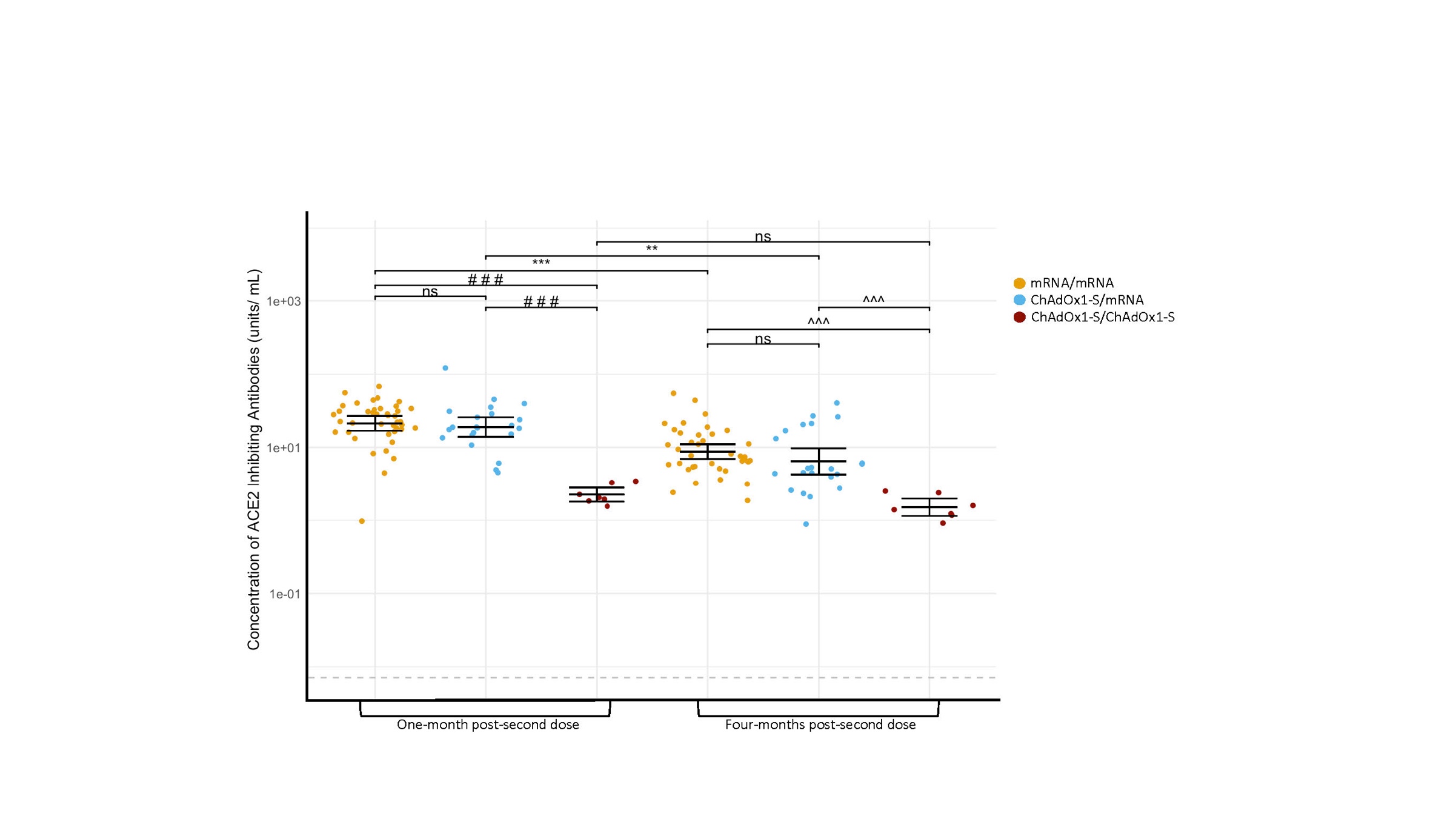


**4g**

**4h**


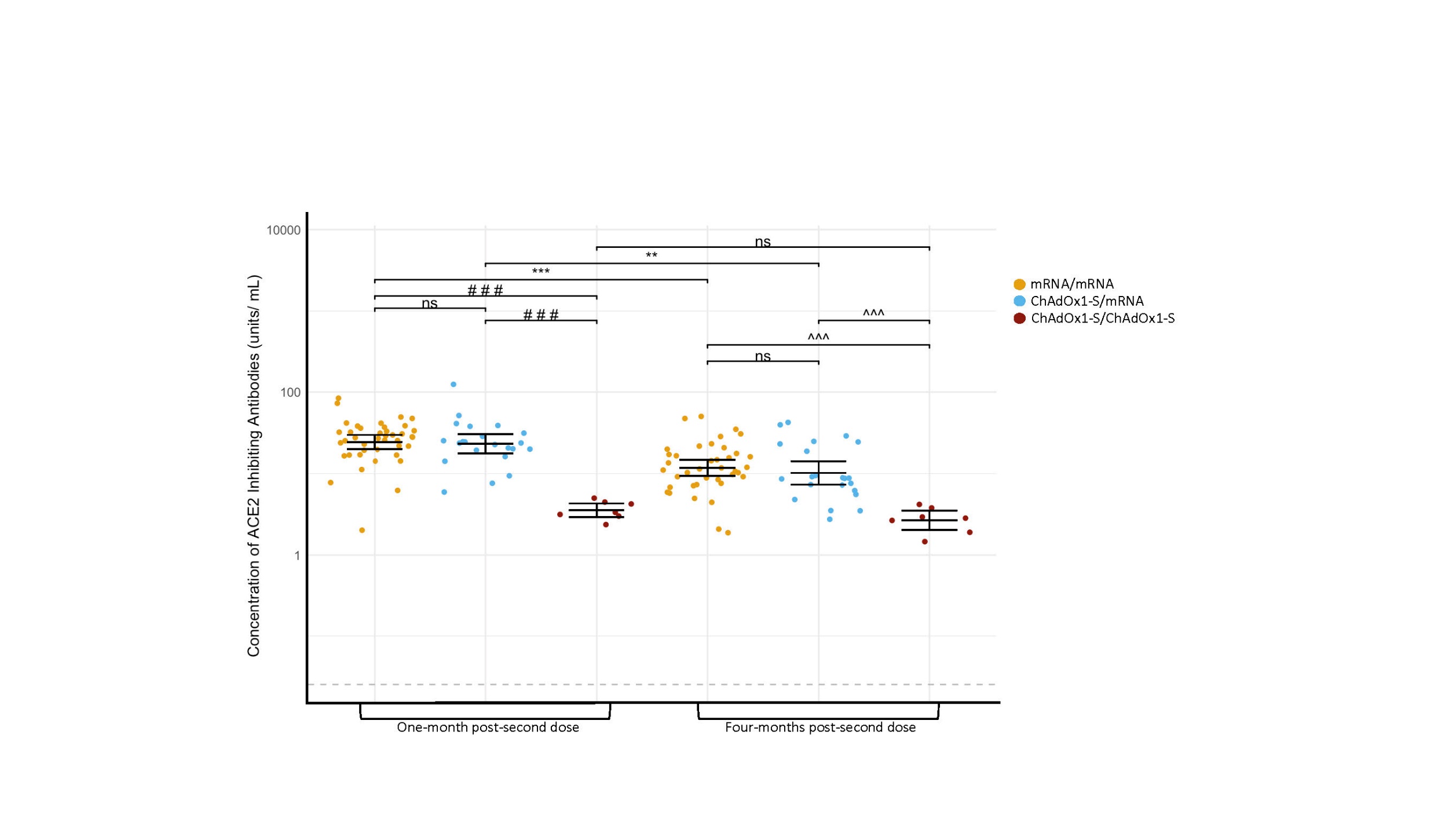


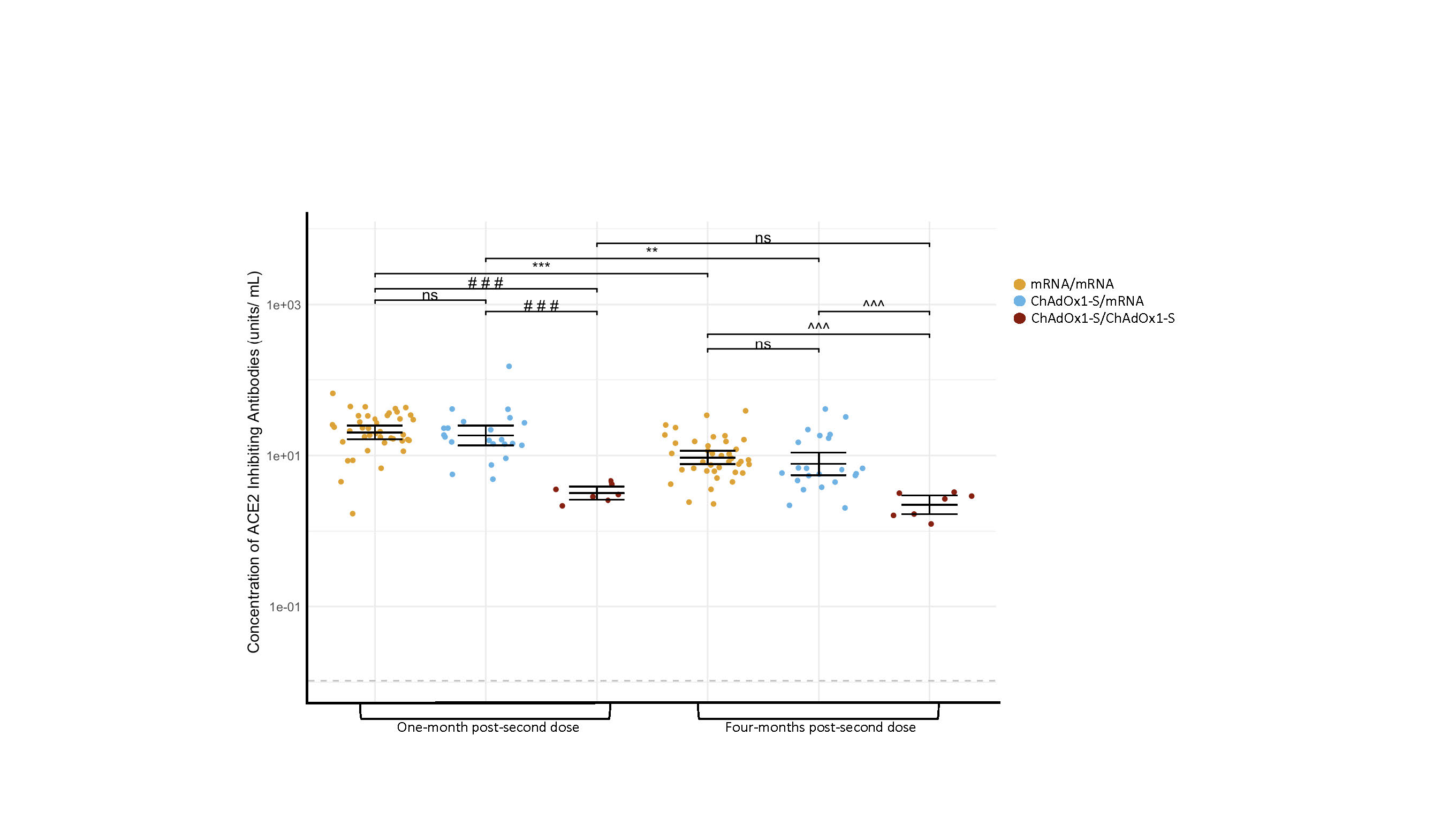


**4i**

**Supplementary fig 4.** **Geometric mean concentrations of ACE2 inhibiting antibodies specific to SARS-CoV-2 spike protein (nine variants) of infection-naïve participants at one- and four-months post-second dose based on vaccine series**. The GMC is represented by the solid line. Data were log_10_ transformed prior to statistical analyses. The grey dashed line represents the lower limit of quantification (LLQ); values below LLQ were assigned half the value for statistical purposes. **(a)** Alpha (B.1.1.7) LLQ = 0.01620 units/mL, **(b)** Beta (B.1.351) LLQ = 0.007624 units/mL, **(c)** Gamma (P.1) LLQ = 0.01439 units/mL, **(d)** Delta (B.1.617.2) LLQ = 0.03334 units/mL, **(e)** Zeta (P.2) LLQ = 0.01655 units/mL, **(f)** Iota (B.1.526.1) LLQ = 0.01761 units/mL, **(g)** Kappa (B.1.617.1) LLQ = 0.007091 units/mL, **(h)** B.1.617 LLQ = 0.02540 units/mL, **(i)** B.1.617.3 LLQ = 0.01044 units/mL.

** *P*<0.01, ****P*< 0.001compared concentrations of protein specific IgG between the same group at one-month and four-months post-second dose (Welch’s t-test, a Bonferroni correction was applied adjusting the P-values by multiplying by the number of comparisons (seven)). ###*P*<0.001, compared concentrations of protein specific IgG between mRNA/mRNA or ChAdOx1-S/mRNA with ChAdOx1-S/ChAdOx1-S at one-month post-second dose (One-way ANOVA, Tukey-Kramer post-hoc). ^^^*P*<0.001, compared concentrations of protein specific IgG between mRNA/mRNA or ChAdOx1-S/mRNA with ChAdOx1-S/ChAdOx1-S at four-months post-second dose (One-way ANOVA, Tukey-Kramer post-hoc. Not significant (ns) *P*>0.05. One-month post-second dose (mRNA/mRNA n=41) (ChAdOx1-S/mRNA n=22) (ChAdOx1-S/ChAdOx1-S n=7), four-months post-second dose (mRNA/mRNA n=39) (ChAdOx1-S/mRNA n=22) (ChAdOx1-S/ChAdOx1-S n=7).

**Supplementary fig 5.** Avidity sample calculations.

**Supplementary fig 6.** Antibody dependent cellular phagocytosis mean phagocytic scores were calculated by multiplying the percentage of bead positive events by the geometric mean fluorescent intensity of the bead positive events. The final score was an average of two scores per sample. Samples were re-analyzed if the percent coefficient of variation between duplicate wells exceeded 20%. The lower limit of quantification (LLQ) was a mean phagocytic score of 300. Scores below the LLQ were assigned a score of 150 for statistical purposes. The LLQ was determined using serum that had an anti-spike protein specific IgG concentration below the ELISA LLQ (2 BAU/mL).

**Supplementary table 1.** T cell participant demographics

| n | 18 |
| --- | --- |
| Age (years)  mean, median (range) | 64.8, 64 (53, 8) |
| Sex  n (%) | Male = 8 (44.4)  Female = 10 (55.6) |
| BMI  Mean, median (range) | 28.9, 28.3 (22.2, 43.6) |
| Ethnicity  n (%) | Chinese = 1 (5.6) |
|  | White and Indigenous = 1 (5.6) |
|  | White = 16 (88.9) |
| Health Status  n (%) | Excellent = 4 (22.2) |
|  | Very Good = 9 (50) |
|  | Good = 4 (22.2) |
|  | Fair = 1 (5.56) |
| Vaccine interval (weeks)  mean, median (range) | 10.5, 10 (9, 14) |
| First vaccine  n | BNT162b2 = 15 |
|  | mRNA-1273 = 0 |
|  | ChAdOx1-S = 3 |
| Second vaccine  n | BNT162b2 = 17 |
|  | mRNA-1273 = 0 |
|  | ChAdOx1-S = 0 |
|  | None = 1 |
| Vaccine series*  (Infection-naïve)  n | mRNA/mRNA = 11 |
|  | ChAdOx1-S/mRNA = 3 |
|  | ChAdOx1-S/ChAdOx1-S = 0 |
| Vaccine series*  (previously infected)  n | mRNA/mRNA = 1 |
|  | ChAdOx1-S/mRNA = 0 |
|  | ChAdOx1-S/ChAdOx1-S = 0 |

*Infection status by four-months post-second dose

**Supplementary table 2**. Infection-naïve versus previously infected participant concentrations of the total absolute avidity levels (TAA) at each visit.

| Comparison | Infection-naïve  Geometric mean (AAU/mL), (95% CI) | Previously infected  Geometric mean (AAU/mL), (95% CI) | *P*-value |
| --- | --- | --- | --- |
| mRNA one-month post-first dose | 43 (23 – 77) | N/A | Groups not compared. |
| ChAdOx1-S one-month post-first dose | N/A | N/A | Groups not compared. |
| mRNA pre-second dose | 28 (15 – 54) | N/A | Groups not compared. |
| ChAdOx1-S pre-second dose | 13 (3 – 50) | N/A | Groups not compared. |
| mRNA/mRNA one-month post-second dose | 825 (601 – 1133) | 2847 (1786 – 4541) | 0.0103 |
| ChAdOx1-S/mRNA one-month post-second dose | 905 (647 – 1265) | N/A | N too small |
| ChAdOx1-S/ ChAdOx1-S one-month post-second dose | 86 (60 – 124) | 354 (107 – 1175) | 0.702 |
| mRNA/mRNA four-months post-second dose | 345 (270 – 441) | 1580 (932 – 2679) | 0.008 |
| ChAdOx1-S/mRNA four-months post-second dose | 304 (220 – 421) | N/A | N too small |
| ChAdOx1-S/ ChAdOx1-S four-months post-second dose | 60 (42 – 87) | 161 (24 – 1069) | 1.0 |

Geometric mean TAA, absolute arbitrary units/ milliliter (AAU/mL), 95% confidence intervals (CI). Statistical analyses were performed on log_10_ transformed data; a Welch’s t-test compared groups.

**Supplementary table 3**. Infection-naïve versus previously infected participant concentrations of the mean phagocytic scores at each visit.

| Comparison | Infection-naive  mean (95% CI) | Previously infected  mean (95% CI) | *P*-value |
| --- | --- | --- | --- |
| Baseline | 150 (150 – 150) | 150 (150 – 150) | Groups not compared. |
| mRNA one-month post-first dose | 301 (119 – 483) | N/A | Groups not compared. |
| ChAdOx1-S one-month post-first dose | N/A | N/A | Groups not compared. |
| mRNA pre-second dose | 299 (101 – 497) | N/A | Groups not compared. |
| ChAdOx1-S pre-second dose | 150 (150 – 150) | N/A | Groups not compared. |
| mRNA/mRNA one-month post-second dose | 2933 (2323 – 3543) | 6897 (4542 – 9252) | 0.001 |
| ChAdOx1-S/mRNA one-month post-second dose | 2483 (1794 – 3171) | N/A | N too small |
| ChAdOx1-S/ ChAdOx1-S one-month post-second dose | 422 (282 – 561) | 1457 (-424 – 3338) | 0.880 |
| mRNA/mRNA four-months post-second dose | 1254 (976 – 1532) | 4374 (2006 – 6740) | 0.007 |
| ChAdOx1-S/mRNA four-months post-second dose | 1102 (806 – 1398) | N/A | N too small |
| ChAdOx1-S/ ChAdOx1-S four-months post-second dose | 293 (193 – 393) | 1069 (-2216 – 4353) | 1.0 |

Mean phagocytic scores, 95% confidence intervals (CI). Statistical analyses were performed on log_10_ transformed data; a Welch’s t-test compared groups.

**Supplementary table 4**. Infection-naïve versus previously infected participant concentrations of ACE2 inhibiting antibodies at each visit.

| SARS-CoV-2 variant | Study Visit | Vaccine series | Infection-naive  GMC (95% CI) | Previously infected  GMC (95% CI) | *P*-value |
| --- | --- | --- | --- | --- | --- |
| Index  (µg/mL) | One-month post-second dose | mRNA/mRNA | 31.3 (23.4 – 41.8) | 65.7 (43.8 – 98.6) | 0.098 |
|  |  | ChAdOx1-S/mRNA | 28.8 (20.3 – 40.9) | N/A | N too small |
|  |  | ChAdOx1-S/ChAdOx1-S | 2.7 (2.3 – 3.3) | 17.0 (3.7 – 78.2) | 0.698 |
|  | Four-months post-second dose | mRNA/mRNA | 10.7 (8.0 – 14.3) | 55.1 (38.7 – 78.4) | < 0.001 |
|  |  | ChAdOx1-S/mRNA | 8.2 (5.4 – 12.6) | N/A | N too small |
|  |  | ChAdOx1-S/ChAdOx1-S | 2.0 (1.5 – 2.5) | 6.0 (1.0 – 36.4) | 1.0 |
| Alpha  B.1.1.7 | One-month post-second dose | mRNA/mRNA | 25.8 (19.9 – 33.6) | 65.4 (43.5 – 98.5) | 0.028 |
|  |  | ChAdOx1-S/mRNA | 22.8 (16.0 – 32.4) | N/A | N too small |
|  |  | ChAdOx1-S/ChAdOx1-S | 2.3 (1.9 – 2.7) | 14.2 (3.2 – 62.4) | 0.653 |
|  | Four-months post-second dose | mRNA/mRNA | 9.2 (7.0 – 12.1) | 58.6 (40.5 – 84.8) | < 0.001 |
|  |  | ChAdOx1-S/mRNA | 6.7 (4.5 – 10.1) | N/A | N too small |
|  |  | ChAdOx1-S/ChAdOx1-S | 1.7 (1.2 – 3.4) | 5.5 (0.9 – 33.7) | 1.0 |
| Beta  B.1.351 | One-month post-second dose | mRNA/mRNA | 12.5 (10.2 – 15.3) | 32.2 (22.4 – 46.2) | 0.012 |
|  |  | ChAdOx1-S/mRNA | 10.8 (7.4 – 15.9) | N/A | N too small |
|  |  | ChAdOx1-S/ChAdOx1-S | 0.8 (0.1 – 4.4) | 4.8 (1.4 – 16.8) | 0.922 |
|  | Four-months post-second dose | mRNA/mRNA | 5.1 (4.1 – 6.4) | 26.0 (15.7 – 43.0) | 0.004 |
|  |  | ChAdOx1-S/mRNA | 4.4 (3.1 – 6.2) | N/A | N too small |
|  |  | ChAdOx1-S/ChAdOx1-S | 0.2 (0.0 – 1.5) | 0.3 (0.0 – 32.1) | 1.0 |
| Gamma  P.1 | One-month post-second dose | mRNA/mRNA | 11.5 (8.8 – 14.9) | 39.6 (26.0 – 60.3) | 0.005 |
|  |  | ChAdOx1-S/mRNA | 11.3 (7.8 – 16.3) | N/A | N too small |
|  |  | ChAdOx1-S/ChAdOx1-S | 1.4 (1.1 – 1.7) | 5.5 (1.4 – 21.0) | 0.957 |
|  | Four-months post-second dose | mRNA/mRNA | 4.4 (3.5 – 5.6) | 31.0 (17.8 – 54.0) | 0.003 |
|  |  | ChAdOx1-S/mRNA | 3.7 (2.6 – 5.4) | N/A | N too small |
|  |  | ChAdOx1-S/ChAdOx1-S | 0.4 (0.1 – 1.4) | 3.5 (0.7 – 17.7) | 0.668 |
| Delta  B.1.617.2 | One-month post-second dose | mRNA/mRNA | 27.0 (22.4 – 32.6) | 61.2 (39.0 – 96.2) | 0.099 |
|  |  | ChAdOx1-S/mRNA | 26.1 (19.5 – 34.9) | N/A | N too small |
|  |  | ChAdOx1-S/ChAdOx1-S | 3.3 (2.4 – 4.4) | 17.2 (6.8 – 43.6) | 0.237 |
|  | Four-months post-second dose | mRNA/mRNA | 13.3 (10.8 – 16.4) | 56.7 (40.4 – 79.6) | < 0.001 |
|  |  | ChAdOx1-S/mRNA | 10.8 (7.6 – 15.6) | N/A | N too small |
|  |  | ChAdOx1-S/ChAdOx1-S | 1.3 (0.3 – 5.7) | 6.7 (2.0 – 23.1) | 0.985 |
| Zeta  P.2 | One-month post-second dose | mRNA/mRNA | 24.6 (19.1 – 31.7) | 42.5 (28.4 – 63.4) | 0.337 |
|  |  | ChAdOx1-S/mRNA | 24.7 (18.7 – 32.7) | N/A | N too small |
|  |  | ChAdOx1-S/ChAdOx1-S | 3.4 (2.8 – 4.1) | 12.0 (4.3 – 33.4) | 0.640 |
|  | Four-months post-second dose | mRNA/mRNA | 11.7 (9.2 – 14.8) | 38.5 (24.9 – 59.5) | 0.001 |
|  |  | ChAdOx1-S/mRNA | 10.1 (7.0 – 14.5) | N/A | N too small |
|  |  | ChAdOx1-S/ChAdOx1-S | 2.7 (2.2 – 3.4) | 4.8 (1.4 – 16.6) | 1.0 |
| Iota  B.1.526.1 | One-month post-second dose | mRNA/mRNA | 22.1 (17.4 – 27.9) | 42.4 (28.5 – 63.0) | 0.149 |
|  |  | ChAdOx1-S/mRNA | 20.6 (15.0 – 28.3) | N/A | N too small |
|  |  | ChAdOx1-S/ChAdOx1-S | 2.2 (1.5 – 3.2) | 11.9 (4.2 – 33.9) | 0.303 |
|  | Four-months post-second dose | mRNA/mRNA | 9.4 (7.2 – 12.3) | 40.0 (28.5 – 56.3) | < 0.001 |
|  |  | ChAdOx1-S/mRNA | 7.2 (4.7 – 11.0) | N/A | N too small |
|  |  | ChAdOx1-S/ChAdOx1-S | 0.9 (0.2 – 4.0) | 4.61 (1.2 – 17.3) | 1.0 |
| Kappa  B.1.617.1 | One-month post-second dose | mRNA/mRNA | 21.3 (17.0 – 26.8) | 42.7 (28.7 – 63.5) | 0.114 |
|  |  | ChAdOx1-S/mRNA | 19.0 (13.9 – 25.8) | N/A | N too small |
|  |  | ChAdOx1-S/ChAdOx1-S | 2.2 (1.8 – 2.8) | 9.4 (3.5 – 25.2) | 0.440 |
|  | Four-months post-second dose | mRNA/mRNA | 8.8 (7.0 – 11.1) | 38.1 (25.4 – 57.0) | 0.001 |
|  |  | ChAdOx1-S/mRNA | 6.5 (4.3 – 9.7) | N/A | N too small |
|  |  | ChAdOx1-S/ChAdOx1-S | 1.5 (1.1 – 2.0) | 4.0 (1.2 – 13.2) | 1.0 |
| B.1.617 | One-month post-second dose | mRNA/mRNA | 24.4 (20.0 – 29.8) | 42.9 (28.9 – 63.7) | 0.265 |
|  |  | ChAdOx1-S/mRNA | 23.1 (17.6 – 30.3) | N/A | N too small |
|  |  | ChAdOx1-S/ChAdOx1-S | 3.5 (2.9 – 4.3) | 12.4 (4.6 – 33.3) | 0.605 |
|  | Four-months post-second dose | mRNA/mRNA | 11.7 (9.4 – 14.7) | 36.2 (25.1 – 52.1) | 0.004 |
|  |  | ChAdOx1-S/mRNA | 10.2 (7.3 – 14.2) | N/A | N too small |
|  |  | ChAdOx1-S/ChAdOx1-S | 2.7 (2.0 – 3.5) | 4.8 (1.3 – 17.9) | 1.0 |
| B.1.617.3 | One-month post-second dose | mRNA/mRNA | 20.2 (16.4 – 24.8) | 52.9 (34.6 – 80.8) | 0.030 |
|  |  | ChAdOx1-S/mRNA | 18.4 (13.5 – 25.0) | N/A | N too small |
|  |  | ChAdOx1-S/ChAdOx1-S | 3.2 (2.6 – 3.9) | 10.0 (3.6 – 27.5) | 0.779 |
|  | Four-months post-second dose | mRNA/mRNA | 9.4 (7.7 – 11.5) | 40.8 (28.4 – 58.7) | < 0.001 |
|  |  | ChAdOx1-S/mRNA | 7.8 (5.5 – 10.9) | N/A | N too small |
|  |  | ChAdOx1-S/ChAdOx1-S | 2.2 (1.7 – 3.0) | 5.9 (1.9 – 18.0) | 1.0 |

Statistical analyses were performed on log_10_ transformed data; a Welch’s t-test was used to compared groups. Geometric mean concentration (GMC), 95% confidence intervals (CI).

**Supplementary table 5**. Univariable linear regression between either concentration spike protein specific IgG, total relative avidity index, Antibody dependent cellular phagocytosis scores, or ACE2 inhibiting antibodies specific to the index virus and participant demographic factors one-month post-second dose. Data were log_10_ transformed prior to statistical analyses. Confidence interval (CI). Coefficients from univariable and multivariable analyses correspond to log_10_ transformed data.

| Visit | Covariate | Coefficient (95% CI) | *P*-value |
| --- | --- | --- | --- |
| Concentration spike protein specific IgG | Age (≥ 70 years old vs. ≤ 69 years old) | -0.069 (-0.309 – 0.172) | 0.572 |
|  | Sex (Male vs. Female) | -0.076 (-0.277 – 0.126) | 0.456 |
|  | Ethnicity (White vs. Non-white) | -0.126 (-0.401 – 0.149) | 0.364 |
|  | BMI (Normal and Underweight combined vs. Overweight and Obese combined) | -0.225 (-0.419 - -0.032) | 0.023 |
|  | Vaccine series (ChAdOx1-S/ ChAdOx1-S vs. mRNA/mRNA and ChAdOx1-S/mRNA combined) | -0.674 (-0.918 - - 0.430) | < 0.001 |
|  | Vaccine interval (≥ 13 weeks vs. < 13 weeks) | -0.317 (-0.607 - -0.027) | 0.033 |
|  | Health status (Excellent vs. Very good/ Good/ Fair/ Mildly poor combined) | -0.371 (-0.562 - -0.179) | < 0.001 |
|  | Infection status (Previously infected vs. Infection-naïve) | 0.242 (-0.039 – 0.524) | 0.091 |
| TRAI | Age (≥ 70 years old vs. ≤ 69 years old) | 0.026 (-0.016 – 0.067) | 0.222 |
|  | Sex (Male vs. Female) | 0.010 (-0.024 – 0.045) | 0.551 |
|  | Ethnicity (White vs. Non-white) | -0.024 (-0.072 – 0.023) | 0.351 |
|  | BMI (Normal and Underweight combined vs. Overweight and Obese combined) | -0.021 (-0.055 – 0.013) | 0.227 |
|  | Vaccine series (ChAdOx1-S/ ChAdOx1-S vs. mRNA/mRNA and ChAdOx1-S/mRNA combined) | -0.060 (-0.108 - -0.012) | 0.014 |
|  | Vaccine interval (≥ 13 weeks vs. < 13 weeks) | 0.048 (-0.003 – 0.098) | 0.064 |
|  | Health status (Excellent vs. Very good/ Good/ Fair/ Mildly poor combined) | -0.032 (-0.067 – 0.003) | 0.073 |
|  | Infection status (Previously infected vs. Infection-naïve) | 0.022 (-0.022 – 0.071) | 0.377 |
| ADCP scores | Age (≥ 70 years old vs. ≤ 69 years old) | 0.051 (-0.155 – 0.256) | 0.627 |
|  | Sex (Male vs. Female) | -0.104 (-0.276 – 0.067) | 0.228 |
|  | Ethnicity (White vs. Non-white) | -0.062 (-0.298 – 0.174) | 0.603 |
|  | BMI (Normal and Underweight combined vs. Overweight and Obese combined) | -0.211 (-0.375 - -0.047) | 0.013 |
|  | Vaccine series (ChAdOx1-S/ ChAdOx1-S vs. mRNA/mRNA and ChAdOx1-S/mRNA combined) | -0.647(-0.845 - -0.449) | < 0.001 |
|  | Vaccine interval (≥ 13 weeks vs. < 13 weeks) | -0.116 (-0.370 – 0.138) | 0.366 |
|  | Health status (Excellent vs. Very good/ Good/ Fair/ Mildly poor combined) | 0.349 (-0.509 - -0.189) | < 0.001 |
|  | Infection status (Previously infected vs. Infection-naïve) | 0.253 (0.014 – 0.491) | 0.038 |
| ACE2 inhibiting antibody concentrations | Age (≥ 70 years old vs. ≤ 69 years old) | 0.028 (-0.242 – 0.297) | 0.839 |
|  | Sex (Male vs. Female) | -0.008 (-0.234 – 0.218) | 0.942 |
|  | Ethnicity (White vs. Non-white) | -0.020 (-0.329 – 0.289) | 0.897 |
|  | BMI (Normal and Underweight combined vs. Overweight and Obese combined) | -0.345 (-0.555 - -0.136) | 0.002 |
|  | Vaccine series (ChAdOx1-S/ ChAdOx1-S vs. mRNA/mRNA and ChAdOx1-S/mRNA combined) | -0.790 (-1.058 - -0.522) | < 0.001 |
|  | Vaccine interval (≥ 13 weeks vs. < 13 weeks) | -0.299 (-0.626 – 0.028) | 0.073 |
|  | Health status (Excellent vs. Very good/ Good/ Fair/ Mildly poor combined) | -0.433 (-0.645 - -0.221), | < 0.001 |
|  | Infection status (Previously infected vs. Infection-naive) | 0.231 (-0.086 – 0.547) | 0.151 |

**Supplementary table 6.** Univariable linear regression between T cell responses and participant demographic factors.

| Study visit | T cell | Covariate | Coefficient (95% CI) | *P*-value |
| --- | --- | --- | --- | --- |
| One-month post-second dose | CD4^+^ | Age | -2.393 (-5.603 – 0.817) | 0.130 |
|  |  | Sex | 0.5005 (-2.211 – 1.285) | 0.266 |
|  |  | BMI | 0.6724 (-1.475 – 2.819) | 0.508 |
|  |  | Dose Interval | -0.553 (-1.248 – 0.143) | 0.109 |
|  | CD8^+^ | Age | -2.140 (-17.151 – 12.871) | 0.761 |
|  |  | Sex | -2.112 (-0.435 – 2.311) | 0.337 |
|  |  | BMI | -4.685 (-14.724 – 5.355) | 0.329 |
|  |  | Dose Interval | -0.796 (-4.049 – 2.456) | 0.603 |
| Four-months post-second dose | CD4^+^ | Age | -11.166 (-30.447 – 8.116) | 0.229 |
|  |  | Sex | 0.0175 (-1.716 – 1.701) | 0.992 |
|  |  | BMI | -0.100 (-8.895 – 8.694) | 0.980 |
|  |  | Dose Interval | -1.216 (-4.262 – 1.829) | 0.398 |
|  | CD8^+^ | Age | 3.992 (-65.139 – 73.124) | 0.901 |
|  |  | Sex | -12.2512 (-0.870 – 1.679) | 0.468 |
|  |  | BMI | -6.667 (-38.199 – 24.866_ | 0.651 |
|  |  | Dose Interval | -0.134 (-11.052 – 10.785) | 0.979 |

The impacts of clinical variables on full-length, spike specific CD4^+^ and CD8^+^ T cell responses were determined using the lm() function (numerical variables) and glm() function (where categorical variables were dummy coded using 0’s and 1’s). Independent regressions were carried out for each variable at one-month post-second dose (n=15) and four-months post-second dose (n=14).

**Supplementary Table 7a**. Multivariable linear regression between ADCP scores and each fractional absolute avidity levels (FAA).

| Visit | FAA | Concentration of S-IgG at each FAA (BAU/ mL)  GMC, 95% CI | Estimate (95% CI) | Adjusted *P*-value |
| --- | --- | --- | --- | --- |
| One-month post-second dose | Total antibodies (0M NH_4_SCN/ 1X PBS) | 1106 (868 – 1410) | 0.586 (0.493 – 0.679) | < 0.001^a^ |
|  | Very low avidity antibodies (< 0.25 M NH_4_SCN) | 132 (96 – 183) | 0.421 (0.319 – 0.523) | < 0.001^b^ |
|  | Low avidity antibodies (0.25M NH_4_SCN) | 190 (149 – 241) | 0.466 (0.355 – 0.577) | < 0.001^b^ |
|  | Low-medium antibodies (0.5M NH_4_SCN) | 226 (177 – 288) | 0.502 (0.403 – 0.602) | < 0.001^b^ |
|  | Medium avidity antibodies (0.75M NH_4_SCN) | 170 (133 – 219) | 0.536 (0.442 – 0.630) | < 0.001^b^ |
|  | Medium-high avidity antibodies (1.0M NH_4_SCN) | 195 (144 – 264) | 0.655 (0.547 – 0.762) | < 0.001^a^ |
|  | High avidity antibodies (1.5M NH_4_SCN) | 70 (51 – 96) | 0.434 (0.324 – 0.545) | < 0.001^a^ |
|  | Very high avidity antibodies (2.0M NH_4_SCN) | 41 (29 – 59) | 0.301 (0.217 – 0.386) | < 0.001^b^ |

a – adjusted for vaccine series and infection status

b – adjusted for vaccines series, infection status and health status

NH_4_SCN: ammonium thiocyanate

S-IgG: anti-spike protein IgG

FAA level: antibody binding strength corresponding to the concentration of NH_4_SCN

Binding antibody units per milliliter (BAU/mL) Geometric mean concentration (GMC), 95% Confidence interval (CI). Data were log_10_ transformed prior to statistical analyses.

**Supplementary table 7b.** Multivariable linear regression analyses ACE2 index vs. fractional absolute avidity levels (FAA).

| Visit | FAA | Concentration of S-IgG at each FAA (BAU/ mL)  GMC, 95% CI | Estimate (95% CI) | Adjusted *P*-value |
| --- | --- | --- | --- | --- |
| One-month post-second dose | Total antibodies (0M NH_4_SCN/ 1X PBS) | 1106 (868 – 1410) | 0.833 (0.703 – 0.963) | < 0.001^c^ |
|  | Very low avidity antibodies (< 0.25 M NH_4_SCN) | 132 (96 – 183) | 0.603 (0.448 – 0.758) | < 0.001^d^ |
|  | Low avidity antibodies (0.25M NH_4_SCN) | 190 (149 – 241) | 0.735 (0.597 – 0.874) | < 0.001^e^ |
|  | Low-medium antibodies (0.5M NH_4_SCN) | 226 (177 – 288) | 0.756 (0.625 – 0.887) | < 0.001^e^ |
|  | Medium avidity antibodies (0.75M NH_4_SCN) | 170 (133 – 219) | 0.823 (0.700 – 0.946) | < 0.001^c^ |
|  | Medium-high avidity antibodies (1.0M NH_4_SCN) | 195 (144 – 264) | 0.887 (0.778 – 0.996) | < 0.001 |
|  | High avidity antibodies (1.5M NH_4_SCN) | 70 (51 – 96) | 0.510 (0.341 – 0.679) | < 0.001^e^ |
|  | Very high avidity antibodies (2.0M NH_4_SCN) | 41 (29 – 59) | 0.392 (0.273 – 0.511) | < 0.001^e^ |

a – adjusted for vaccine series and infection status

c – adjusted for vaccine series

d – adjusted for vaccine series and health status

e – adjusted for vaccine series and BMI

f – adjusted for vaccine series, vaccine interval and infection status

NH_4_SCN: ammonium thiocyanate

S-IgG: anti-spike protein IgG

FAA: antibody binding strength corresponding to the concentration of NH_4_SCN

Binding antibody units per milliliter (BAU/mL) Geometric mean concentration (GMC), 95% Confidence interval (CI). Data were log_10_ transformed prior to statistical analyses.

**Supplementary table 8a.** Spearman correlation between anti-spike protein IgG and antibody function.

| Visit | Participant group | Comparison | r | *P*-value |
| --- | --- | --- | --- | --- |
| One-month post-second dose | All participants | Anti-S IgG vs. TRAI | 0.220 | 0.386 |
|  |  | Anti-S IgG vs. ADCP mean phagocytic scores | 0.818 | < 0.001 |
|  |  | Anti-S IgG vs. ACE2 inhibiting antibody concentration (index virus) | 0.824 | < 0.001 |
|  | mRNA/mRNA | Anti-S IgG vs. TRAI | -0.058 | 0.386 |
|  |  | Anti-S IgG vs. ADCP mean phagocytic scores | 0.699 | < 0.001 |
|  |  | Anti-S IgG vs. ACE2 inhibiting antibody concentration (index virus) | 0.774 | <0.001 |
|  | ChAdOx1-S/mRNA | Anti-S IgG vs. TRAI | 0.094 | 1.0 |
|  |  | Anti-S IgG vs. ADCP mean phagocytic scores | 0.729 | 0.001 |
|  |  | Anti-S IgG vs. ACE2 inhibiting antibody concentration (index virus) | 0.776 | < 0.001 |
|  | ChAdOx1-S/ChAdOx1-S | Anti-S IgG vs. TRAI | -0.750 | 0.530 |
|  |  | Anti-S IgG vs. ADCP mean phagocytic scores | 0.821 | 0.273 |
|  |  | Anti-S IgG vs. ACE2 inhibiting antibody concentration (index virus) | 0.214 | 1.0 |
| Four-months post-second dose | All participants | Anti-S IgG vs. TRAI | 0.411 | 0.002 |
|  |  | Anti-S IgG vs. ADCP mean phagocytic scores | 0.908 | < 0.001 |
|  |  | Anti-S IgG vs. ACE2 inhibiting antibody concentration (index virus) | 0.924 | < 0.001 |
|  | mRNA/mRNA | Anti-S IgG vs. TRAI | 0.191 | 1.0 |
|  |  | Anti-S IgG vs. ADCP mean phagocytic scores | 0.765 | < 0.001 |
|  |  | Anti-S IgG vs. ACE2 inhibiting antibody concentration (index virus) | 0.818 | < 0.001 |
|  | ChAdOx1-S/mRNA | Anti-S IgG vs. TRAI | 0.089 | 1.0 |
|  |  | Anti-S IgG vs. ADCP mean phagocytic scores | 0.910 | < 0.001 |
|  |  | Anti-S IgG vs. ACE2 inhibiting antibody concentration (index virus) | 0.874 | < 0.001 |
|  | ChAdOx1-S/ChAdOx1-S | Anti-S IgG vs. TRAI | -0.893 | 0.098 |
|  |  | Anti-S IgG vs. ADCP mean phagocytic scores | 0.216 | 1.0 |
|  |  | Anti-S IgG vs. ACE2 inhibiting antibody concentration (index virus) | -0.393 | 1.0 |

Anti-S IgG: anti-spike protein IgG

TRAI: total relative avidity index

ADCP: antibody dependent cellular phagocytosis

ACE2: angiotensin-converting enzyme 2

Data were log_10_ transformed prior to statistical analyses; a Bonferroni correction was applied adjusting the P-values by multiplying by the number of comparisons (eight).

**Supplementary table 8b.** Spearman correlation between total absolute avidity levels (TAA) and antibody function.

| Visit | Participant group | Comparison | r | *P*-value |
| --- | --- | --- | --- | --- |
| One-month post-second dose | All participants | TAA vs. ADCP mean phagocytic scores | 0.878 | < 0.001 |
|  |  | TAA vs. ACE2 inhibiting antibody concentration (index virus) | 0.829 | < 0.001 |
|  | mRNA/mRNA | TAA vs. ADCP mean phagocytic scores | 0.774 | < 0.001 |
|  |  | TAA vs. ACE2 inhibiting antibody concentration (index virus) | 0.764 | < 0.001 |
|  | ChAdOx1-S/mRNA | TAA vs. ADCP mean phagocytic scores | 0.836 | < 0.001 |
|  |  | TAA vs. ACE2 inhibiting antibody concentration (index virus) | 0.797 | < 0.001 |
|  | ChAdOx1-S/ChAdOx1-S | TAA vs. ADCP mean phagocytic scores | 0.857 | 0.190 |
|  |  | TAA vs. ACE2 inhibiting antibody concentration (index virus) | 0.286 | 1.0 |
| Four-months post-second dose | All participants | TAA vs. ADCP mean phagocytic scores | 0.875 | < 0.001 |
|  |  | TAA vs. ACE2 inhibiting antibody concentration (index virus) | 0.895 | < 0.001 |
|  | mRNA/mRNA | TAA vs. ADCP mean phagocytic scores | 0.687 | < 0.001 |
|  |  | TAA vs. ACE2 inhibiting antibody concentration (index virus) | 0.689 | < 0.001 |
|  | ChAdOx1-S/mRNA | TAA vs. ADCP mean phagocytic scores | 0.854 | < 0.001 |
|  |  | TAA vs. ACE2 inhibiting antibody concentration (index virus) | 0.893 | < 0.001 |
|  | ChAdOx1-S/ChAdOx1-S | TAA vs. ADCP mean phagocytic scores | 0.450 | 1.0 |
|  |  | TAA vs. ACE2 | -0.071 | 1.0 |

TAA: total absolute avidity levels

ADCP: antibody dependent cellular phagocytosis

ACE2: angiotensin-converting enzyme 2

Data were log_10_ transformed prior to statistical analyses; a Bonferroni correction was applied adjusting the P-values by multiplying by the number of comparisons (eight).

**Supplementary table 9**. The total number and proportion of participants that achieved suggested correlates of protection.

|  | Correlate of protection (BAU/ mL) | Vaccine series | One-month post-second dose  n (%) | Four-months post-second dose  n (%) |
| --- | --- | --- | --- | --- |
| All participants | 60 and greater | mRNA/mRNA | 46 (100) | 43 (97.7) |
|  |  | ChAdOx1-S/mRNA | 23 (100) | 23 (100) |
|  |  | ChAdOx1-S/ ChAdOx1-S | 11 (100) | 7 (63.6) |
|  | 100 and greater | mRNA/mRNA | 46 (100) | 43 (97.7) |
|  |  | ChAdOx1-S/mRNA | 23 (100) | 23 (100) |
|  |  | ChAdOx1-S/ ChAdOx1-S | 10 (90.9) | 5 (50) |
|  | 154 and greater | mRNA/mRNA | 46 (100) | 42 (95.5) |
|  |  | ChAdOx1-S/mRNA | 23 (100) | 21 (91.3) |
|  |  | ChAdOx1-S/ ChAdOx1-S | 8 (72.7) | 3 (27.3) |
| Infection-naive participants only | 60 and greater | mRNA/mRNA | 40 (97.6) | 37 (97.4) |
|  |  | ChAdOx1-S/mRNA | 22 (100) | 22 (100) |
|  |  | ChAdOx1-S/ ChAdOx1-S | 7 (100) | 4 (57.1) |
|  | 100 and greater | mRNA/mRNA | 40 (97.6) | 37 (97.4) |
|  |  | ChAdOx1-S/mRNA | 22 (100) | 22 (100) |
|  |  | ChAdOx1-S/ ChAdOx1-S | 6 (85.7) | 3 (42.9) |
|  | 154 and greater | mRNA/mRNA | 40 (97.6) | 36 (94.7) |
|  |  | ChAdOx1-S/mRNA | 22 (100) | 20 (90.9) |
|  |  | ChAdOx1-S/ ChAdOx1-S | 4 (57.1) | 2 (28.6) |

**Supplementary table 10**. Description of health status levels.

| Health Status | Definition |
| --- | --- |
| Excellent | You are robust, active, energetic and motivated. You exercise regularly and categorize yourself as very fit. |
| Very Good | You do not have current medical problems. You exercise occasionally or seasonally. |
| Good | Your medical problems are well controlled. You are not regularly active beyond routine walking. |
| Fair | You are not dependent on others for daily help, but often your medical problems limit activities. |
| Mildly poor | You need help with some outside activities like shopping and some activities indoors like meal preparation and housework. |
| Poor | You need help with all outside activities and with keeping house. |
| Very poor | Completely dependent for personal care, from whatever cause (physical or cognitive). |

**Supplementary table 11.** Activation induced marker (AIM) assay markers.

| Target | Conjugated  fluorochrome | Clone | Company | Catalogue number | Concentration |
| --- | --- | --- | --- | --- | --- |
| CXCR5 (CD185) | BUV496 | RF8B2 | BD | 741115 | 1/100 |
| PD-1 (CD279) | BUV737 | EH12.1 | BD | 612791 | 1/100 |
| CXCR3 (CD183) | BV421 | G025H7 | BioLegend | 353716 | 1/100 |
| CD3 | BV510 | UCHT1 | BD | 563109 | 1/200 |
| CCR4 (CD194) | BV605 | L291H4 | BioLegend | 359418 | 1/200 |
| CD69 | BV711 | FN50 | BioLegend | 310944 | 1/100 |
| CCR6 (CD196) | BV786 | G034E3 | BioLegend | 353422 | 1/100 |
| CD8 | BB515 | RPA-T8 | BD | 564526 | 1/200 |
| CD4 | BB700 | SK3 | BD | 566392 | 1/200 |
| OX40 (CD134) | PE | L106 | BD | 340420 | 1/10 |
| CD39 | PEDazzle594 | A1 | BioLegend | 328224 | 1/100 |
| CD25 | PECy7 | M-A251 | BD | 557741 | 1/40 |
| 4-1BB (CD137) | APC | 4B4-1 | BioLegend | 309810 | 1/100 |
